# Mesenchymal stromal cells induce neutrophil aggregation and extracellular vesicle storms for systemic lupus erythematosus

**DOI:** 10.1038/s41392-025-02442-1

**Published:** 2025-10-13

**Authors:** Qianmin Ou, Luhan Niu, Dandan Wang, Genhong Yao, Qianhui Ren, Zhengshi Li, Xueli Mao, Wei Teng, Zetao Chen, Andy Peng Xiang, Songtao Shi, Lingyun Sun

**Affiliations:** 1 South China Center of Craniofacial Stem Cell Research, Hospital of Stomatology, Sun Yat-sen University, Guangdong Provincial Key Laboratory of Stomatology, Guangzhou, China; 2 Department of Rheumatology and Immunology, Nanjing Drum Tower Hospital, Affiliated Hospital of Medical School, Nanjing University, Nanjing, China; 3 Center for Stem Cell Biology and Tissue Engineering, Key Laboratory for Stem Cells and Tissue Engineering, Ministry of Education, Sun Yat-Sen University, Guangzhou, China

**Keywords:** Mesenchymal stem cells, Immunological disorders

## Abstract

Mesenchymal stromal cell (MSC) transplantation has achieved significant clinical benefits for many diseases, such as systemic lupus erythematosus (SLE) and inflammatory diseases. However, the detailed therapeutic mechanism of MSCs is not fully understood. Here, in the SLE treatment, we show that MSC transplantation triggers recipient bone marrow neutrophil aggregation to generate an endogenous extracellular vesicle (EV) storm in the circulation via the TNFα/ICAM-1/Rab11b axis. Interestingly, blockade of the EV storm abolishes the MSC-mediated therapeutic effect for SLE. The level of EV storm is positively associated with the therapeutic effect of MSCs in SLE patients. Mechanistically, aggregated neutrophils-derived EV storms equalize Th17 and T-regulatory (Treg) cells to promote immune tolerance and disease remission *via* the DHA/LILRB4/STAT5/STAT3 pathway in the MSC treatment for SLE. Taken together, our findings reveal a new immune-modulating function of MSCs through the induction of endogenous neutrophil aggregation in the bone marrow, which results in the secretion of EV storms for immune tolerance in SLE mice and patients. In addition, this study revealed a previously unknown role of the recipient EV storm in determining the therapeutic effect of MSC in SLE, and the recipient EV storm can be used to predict the therapeutic efficacy of MSCs in SLE therapy.

## Introduction

Mesenchymal stromal cell (MSC) transplantation has been widely used to treat a variety of diseases, such as graft-vs-host disease, systemic lupus erythematosus (SLE) and liver fibrosis.^1,2^ The safety and efficacy of MSC transplantation have been widely demonstrated in previous studies.^2–4^ In previous studies, various therapeutic mechanisms of MSC therapy have been proposed, such as (1) paracrine regulation of immunomodulatory, angiogenic, antiapoptotic and antioxidative effects; (2) epigenetic regulation for immune cell and tissue cells; (3) cell‒cell contact for the regulation of target cells; (4) mitochondrial transfer for the regulation of target cells; and (5) cell death-mediated regulation for immune homeostasis.^5–7^ These mechanisms are mainly focus on the functions and properties of MSC. However, the MSC are living cells, and it is unknown whether recipient responses contribute to the MSC function on related disease treatment, such as SLE. Nowadays, MSC therapy is gradually being applied to clinical patients. The US Food and Drug Administration (FDA) approved the first MSC product (Remestemcel-L) for pediatric acute steroid-refractory graft-versus-host-disease treatment in 2014, which suggests that the safety and efficacy of MSC transplantation are increasingly being recognized.^8^

Aggregation is a ubiquitous biological phenomenon. For instance, honeybees can release a pheromones and recruit nearby honeybees, then they will aggregate together and then kill invading predators by thermo-balling.^9,10^ Therefore, the phenomenon of aggregation is an important way for the organism to protect itself. Besides, cell aggregation is a biological mechanism in the development of various organs, such as the liver, intestine and lymph nodes.^9,10^ The cell aggregation is necessary for the organ’s development and the impaired cell aggregation will delay the organs development.^9,10^ Interestingly, cell aggregation is not a simple addition of cellular functions, which increases adaptivity to environmental challenges during organ development.^11,12^ In addition, the aggregation of specific cells is an important means for an organism to protect itself. Neutrophils are the key cells of innate immune response in the circulation and are important for immune homeostasis.^13,14^ Neutrophil aggregation (swarming) is a special feature of neutrophil activation in response to a variety of inflammatory stimuli and sterile tissue damage.^15,16^ In general, the process of neutrophils aggregation includes: (1) neutrophils aggregation initiation through tissue injury and amplification through secondary cell death; (2) Swarm initiation through intercellular signal relay; (3) neutrophils aggregation and resolution.^16^ Upon infection, substantial numbers of neutrophils migrate and aggregate at the inflammatory site, where they perform anti-inflammatory functions.^16^ To date, researchers have not determined whether MSC transplantation results in any cell aggregation.

An imbalance between Th17 and T regulatory (Treg) cells is involved in the onset and development of organ inflammation in SLE.^17^ In general, The high ratio of Th17 cells can intensify the inflammatory response, while the high ratio of Treg cells can alleviate the inflammatory response.^17^ Thus, rebalancing the Th17/Treg cell ratio is crucial for SLE treatment, which is one of the common mechanisms of the MSC treatment in SLE and other disease.^18^ Previous studies showed that docosahexaenoic acid (DHA) is an important omega-3 fatty acid with immunomodulatory functions, which can exert the function of maintaining the immune homeostasis.^19,20^ Leukocyte immunoglobulin-like receptor B4 (LILRB4) is an immune inhibitory receptor that can support tumor cell infiltration into tissues and suppress T-cell activity.^21^ Recent studies suggest that LILRB4 is a compelling target for cancer treatment that can ameliorate the LILRB4-induced immune-suppressive microenvironment.^22,23^ Gp49B (the mouse counterpart of LILRB4) is expressed in the plasma cells and plasmablasts of SLE mice but not in those of control C57BL/6 mice. The expression level is positively correlated with the anti-dsDNA IgG titer in serum.^24^ However, the function of DHA on the balance of the Th17/Treg cell ratio is unknown.

In this study, we show that allogeneic MSC transplantation induces recipient neutrophil aggregation in the bone marrow, accompanied by the generation of endogenous extracellular vesicle (EV) storms in the circulation of SLE mice and human patients *via* the TNFα/ICAM-1/Rab11b axis. In addition, the MSC-triggered EV storms in SLE mice are cell type-specific and dose-dependent. The EV storm is necessary for the MSC treatment on SLE, and the blockade of the EV storm can abolish the MSC-mediated therapeutic effect for SLE mice. MSC induced aggregated neutrophil-derived EV storms, which contain high levels of DHA, exert the vital function of rebalancing the Th17/Treg ratio *via* the LILRB4/STAT5/STAT3 pathway. The inhibition of DHA content in EV storm could impair the therapeutic effect of EV storm in SLE mice. Interestingly, the level of the EV storm is positively correlated with the efficacy of MSC transplantation in SLE patients, suggesting that the EV storm can be used as an early predictor of the effectiveness of MSCs for SLE. This study revealed a previously unreported mechanism of the recipient EV storm in determining the therapeutic effect of MSCs in SLE.

## Results

### MSC transplantation triggers EV storms in a cell type- and dose-dependent manner

**Fig. 1:**
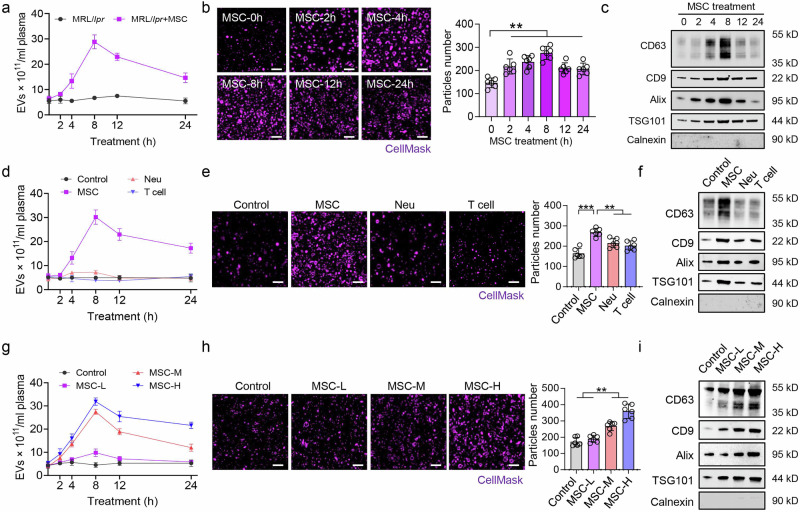
MSC-induced recipient EV storm is cell type-specific and dose dependent. **a** ZetaView assay showed that the number of EVs in the blood of MRL/*lpr* mice increased after MSC transplantation, n = 3 per group. **b** Structured illumination microscopy (SIM) revealed that the number of EVs was increased in the blood of MRL/*lpr* mice at 2, 4, 8, 12, and 24 h postMSC transplantation (n = 6). Scale bar = 1 μm. **c** Western blotting demonstrated increased expression of the EV markers CD63, CD9, Alix and TSG101 at 4 and 8 h postMSC transplantation (n = 3). **d** A ZetaView assay indicated that MSC transplantation promoted the production of EVs in the blood of MRL/*lpr* control mice, whereas neutrophil or T-cell infusion failed to do so. Neu, neutrophils, n = 3. **e**, **f** SIM and western blotting revealed that neutrophil and T-cell infusion failed to enhance EV production or promote the expression of EV markers in the blood of MRL/*lpr* mice. n = 6, Scale bar = 1 μm (**e**), n = 3 (**f**). **g** ZetaView assays revealed that a moderate number of MSCs (1 × 10^6^_;_ MSC-M) and a high number of MSCs (2 × 10^6^; MSC-H) increased the number of EVs in the blood of MRL/*lpr* mice, but a low concentration of MSCs (0.1 × 10^6^; MSC-L) did not have that effect, n = 3. **h**, **i** SIM and western blotting revealed that both MSC-Ms and MSC-Hs increased EV production in the blood of MRL/*lpr* mice at 8 h postMSC transplantation. Scale bar = 1 μm, n = 6 for (**h**), n = 3 for (**i**). **p < 0.01; ***p < 0.001

EVs are important media for cell communication and tissue hemostasis and exert multiple biological effects.^25,26^ To investigate the potential recipient response during MSC transplantation, we analyzed the EV concentration in the blood of MRL/*lpr* mice, a classic SLE model, after treatment with MSCs. ZetaView and structured illumination microscopy (SIM) analysis revealed a significant increase in the number of EVs at 8 h after MSC transplantation (defined as an EV storm**;** Fig. 1a, b, Supplementary Fig. 1a). The expression levels of the EV markers CD63, CD9, Alix, and TSG101 were increased at 8 h postMSC transplantation (Fig. 1c). We also observed EV storms in imiquimod (IMQ)-induced SLE mice (Supplementary Fig. 1b–d). Moreover, MSC transplantation induced a lower level of EV storm in wild-type mice than in SLE mice (Supplementary Fig. 1e).

Interestingly, compared with MSC-induced EV storm, when the same number of neutrophils or T cells were infused into MRL/*lpr* mice, they failed to induce an EV storm (Fig. 1d–f). Furthermore, we examined the effects of the MSC concentration on the generation of EV storms in MRL/*lpr* mice and found that intravenous infusion of 1 × 10^6^ MSCs (MSC-M) or 2 × 10^6^ MSCs (MSC-H) induced an EV storm in the blood of MRL/*lpr* mice, but intravenous infusion of 0.1 × 10^6^ MSCs (MSC-L) failed to do so (Fig. 1g–i). These data suggest that MSC-triggered EV storms in SLE mice are cell type-specific and dose dependent.

To analyze the function of the EV storm in MSC-treated SLE mice, we used GW4869, an inhibitor of exosome biogenesis, to inhibit the formation of EVs. Treatment with GW4869 inhibited the EV storm in the blood of MRL/*lpr* mice treated with MSCs (Supplementary Fig. 2a). Interestingly, GW4869 also impaired the inhibitory effects of MSCs on the concentrations of serum antinuclear antibodies (ANAs) and blood urea nitrogen (BUN) in MRL/*lpr* mice (Supplementary Fig. 2b). Previous studies have reported that MSC-derived EVs (MEVs) also alleviate SLE^27,28^; thus, we tested whether MEVs could induce an EV storm in SLE mice. The EV concentration was increased at 8 hours in MRL/*lpr* mice after MEV treatment (Supplementary Fig. 2c). Notably, GW4869 inhibited the level of EV storm and the therapeutic function of MEV in MRL/*lpr* mice (Supplementary Fig. 2c, d). Therefore, these results indicate that the level of EV storm is related to the therapeutic efficacy in SLE mice.

### EV storms mainly originate from recipient bone marrow neutrophils

To assess the source of EV storms, we infused PKH26-labeled MSCs (PKH26-MSCs) into GFP-expressing mice (Supplementary Fig. 3a). Nano-flow cytometry (nFCM) analysis revealed that the number of PKH26+ EVs in the blood was not significantly greater than that in the control group at 8 h post-PKH26-MSC infusion (Supplementary Fig. 3a). However, the number of GFP^+^ EVs increased when PKH26-MSCs were infused into the GFP mice (Supplementary Fig. 3a). These results indicate that MSC-evoked EV storms in the blood may originate from endogenous recipient cells.

**Fig. 2:**
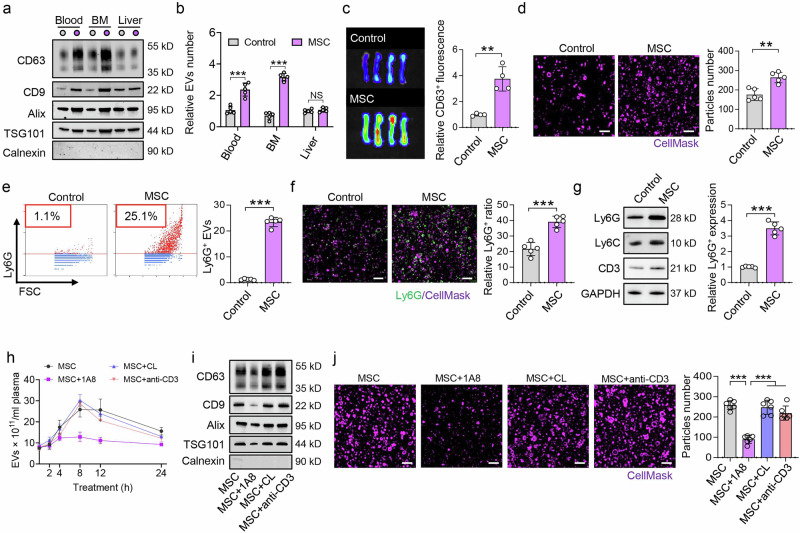
EV storms originate from bone marrow neutrophils. **a** Western blotting revealed that MSC transplantation increased the expression of the EV markers CD63, CD9, Alix, and TSG101 in the bone marrow (BM) of MRL/*lpr* mice, n = 3 per group. **b** ZetaView assay results indicating that EV levels were increased in the BM and blood of MRL/*lpr* mice at 8 h postMSC transplantation, n = 5. **c** IVIM analysis revealed that CD63 fluorescence was increased in the BM of MRL/*lpr* mice at 8 h postMSC transplantation (n = 4). **d** SIM revealed that the number of EVs in the BM was increased at 8 h postMSC transplantation (n = 5). Scale bar = 1 μm. **e** Nanoflow cytometry (nFCM) and **f** SIM analyses revealed that the number of Ly6G^+^ EVs was increased in the BM at 8 h postMSC transplantation (n = 5). Scale bar = 1 μm. **g** Western blotting revealed that the expression of Ly6G was increased in EVs in the blood at 8 h postMSC transplantation (n = 5). **h** 1A8 (anti-Ly6G antibody) treatment inhibited the increase in blood EVs in MRL/*lpr* mice at 8 h postMSC transplantation, whereas clodronate liposome (CL) and anti-CD3 antibody treatment did not have this effect (n = 3). **i**, **j** Treatment with 1A8 inhibited the expression of EV markers and EV production in the blood of MRL/*lpr* mice at 8 h postMSC transplantation. n = 3 (**i**), n = 5; scale bar = 1 μm (**j**). NS not significant; **p < 0.01; ***p < 0.001

To further explore the origins of EV storms, we analyzed EV levels in different organs of SLE mice treated with MSCs. Western blotting and ZetaView results revealed that EV levels were significantly increased in the bone marrow and blood of MRL/*lpr* mice at 8 h postMSC transplantation (Fig. 2a, b). CD63^+^ fluorescence was also increased in the bone marrow of MRL/*lpr* mice at 8 h postMSC transplantation (Fig. 2c). In addition, SIM analysis revealed that the number of EVs increased in the bone marrow at 8 h postMSC transplantation (Fig. 2d). However, the EV levels in the liver, spleen, lung, and heart of MRL/*lpr* mice were not significantly changed at 8 h postMSC transplantation (Fig. 2a, b, Supplementary Fig. 3b–g).

To examine the cellular origins of EV storms, we collected EVs from the bone marrow of MRL/*lpr* mice at 8 h postMSC transplantation and assessed their cellular origins via different cell markers. The EVs highly expressed the neutrophil-specific marker Ly6G (Fig. 2e–g) but expressed low levels of the macrophage marker F4/80, the T-cell marker CD3, the B-cell marker B220, the mesenchymal stromal cell marker CD90, and the endothelial cell marker CD31 (Supplementary Fig. 3h). We subsequently used 1A8 (an anti-neutrophil antibody), clodronate liposomes (CLs), and an anti-CD3 antibody to deplete neutrophils, macrophages, and CD3^+^ T cells, respectively (Supplementary Fig. 3i).^29,30^ The results showed that 1A8 pretreatment inhibited EV storm in the blood of MRL/*lpr* mice at 8 h postMSC transplantation, whereas pretreatment with CL or anti-CD3 antibody did not prevent the generation of EV storms (Fig. 2h–j). Taken together, these results indicate that MSC-induced EV storms mainly originate from recipient neutrophils in the bone marrow.

### MSCs induce neutrophil aggregation in the bone marrow of SLE mice

**Fig. 3:**
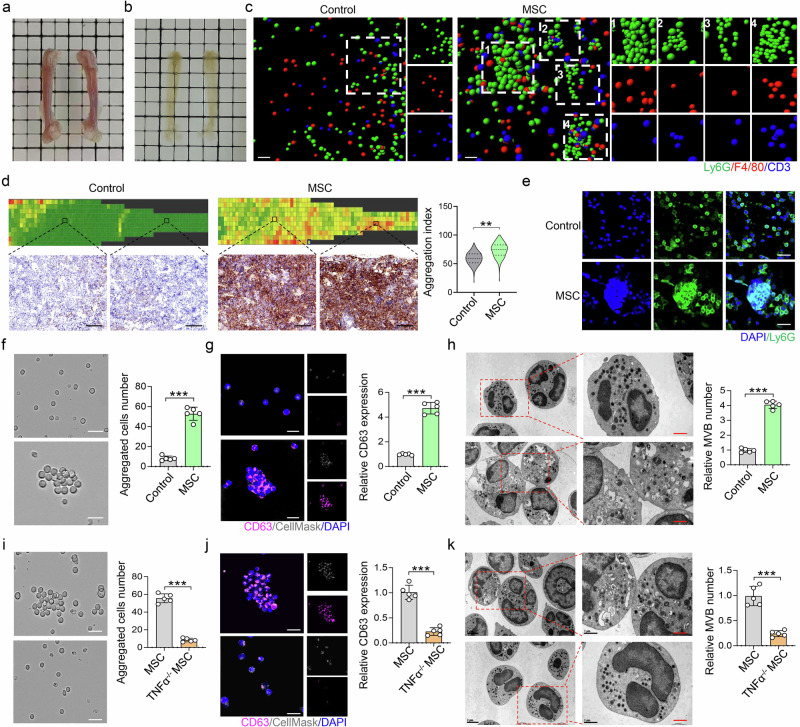
MSC transplantation induces neutrophil aggregation in the bone marrow through TNFα. The morphology of the bone marrow of MRL/*lpr* mice before (**a**) and after **b** tissue clearing, Scale bar = 0.25 cm. **c** Tissue transparency experiments revealed that MSC transplantation induced Ly6G^+^ neutrophil aggregation in the BM of MRL/*lpr* mice, but F4/80^+^ macrophages and CD3^+^ T cells did not aggregate, scale bar = 200 μm. **d** HALO proximity analysis indicated that MSC transplantation induced neutrophil aggregation in the BM at 8 h postMSC transplantation. Scale bar = 40 μm, n = 3 per group. **e** BM smears revealed that MSC transplantation induced Ly6G^+^ neutrophil aggregation in MRL/*lpr* mice, n = 3. Scale bar = 20 μm. **f** MSC supernatant induced neutrophil aggregation in vitro, n = 5. Scale bar = 20 μm. **g** MSC supernatant induced cell aggregation and CD63 expression in neutrophils, n = 5. Scale bar = 10 μm. **h** TEM revealed that MSC supernatant induced cell aggregation and promoted multivesicular body (MVB) formation in neutrophils (n = 5). Scale bar = 1 μm. **i**–**k** Neutrophil aggregation, CD63 expression and MVB formation were decreased when neutrophils were stimulated with the supernatant of TNFα^−/−^ MSCs (n = 5). Scale bar = 20 μm (**i**), scale bar = 10 μm (**j**), scale bar = 1 μm (**k**). **p < 0.01; ***p < 0.001

To gain further insight into the effects of MSC transplantation on recipient neutrophils, we evaluated the proliferation and apoptosis of neutrophils in the bone marrow of MRL/*lpr* mice after MSC transplantation. Interestingly, we found that MSC transplantation did not affect the proliferation or apoptosis of neutrophils (Supplementary Fig. 4a). We then used tissue-clearing technology and three-dimensional imaging to assess recipient neutrophils in the bone marrow (Fig. 3a, b). Surprisingly, we found that MSC transplantation promoted neutrophil aggregation in the bone marrow, but no aggregation of macrophages or T cells was observed (Fig. 3c). In addition, we used tissue microarray analysis with HALO software to evaluate the degree of neutrophil aggregation. The aggregation index (AI), represented by the Ly6G signal ratio in each microregion, reflects the degree of neutrophil aggregation. MSC transplantation increased the AI value of neutrophils in the bone marrow of MRL/*lpr* mice (Fig. 3d). A bone marrow smear assay also confirmed that MSC transplantation promoted recipient neutrophil aggregation in the bone marrow (Fig. 3e).

To confirm that MSCs secrete soluble factors to induce neutrophil aggregation, we collected the supernatant from culture-expanded MSCs. MSC supernatant promoted neutrophil aggregation in vitro (Fig. 3f, g) and activated CD63 expression in neutrophils (Fig. 3g). Transmission electron microscopy (TEM) further confirmed that the MSC supernatant facilitated multivesicular body (MVB) formation in neutrophils (Fig. 3h).

TNFα can promote the aggregation of primary hepatocytes to form 3D hepatocytes,^31^ and the overexpression of TNF in transgenic mice leads to lymphoid aggregation in the bone marrow.^32^ Thus, we hypothesized that TNFα plays an important role in MSC-induced neutrophil aggregation. We showed that the supernatant of TNFα knockout MSCs (TNFα^−/−^ MSCs) failed to promote neutrophil aggregation, CD63 expression and MVB formation in neutrophils (Fig. 3i–k). TNFα^−/−^ MSC transplantation failed to promote bone marrow neutrophil aggregation and induce EV storms in the blood of MRL/*lpr* mice (Supplementary Fig. 4b–e). The blood EVs isolated from MSCs and TNFα^−/−^ MSC-treated MRL/*lpr* mice were not significantly different in terms of mean diameter, zeta potential or morphology (Supplementary Fig. 4f, g). Moreover, TNFα treatment (50 ng/ml; 8 h) promoted EV production by neutrophils in vitro (Supplementary Fig. 4h). These data suggest that TNFα is required for MSC-induced neutrophil aggregation and EV storm generation in MRL/*lpr* mice.

### Neutrophil aggregation releases EVs *via* the ICAM1/Rab11b pathway

**Fig. 4:**
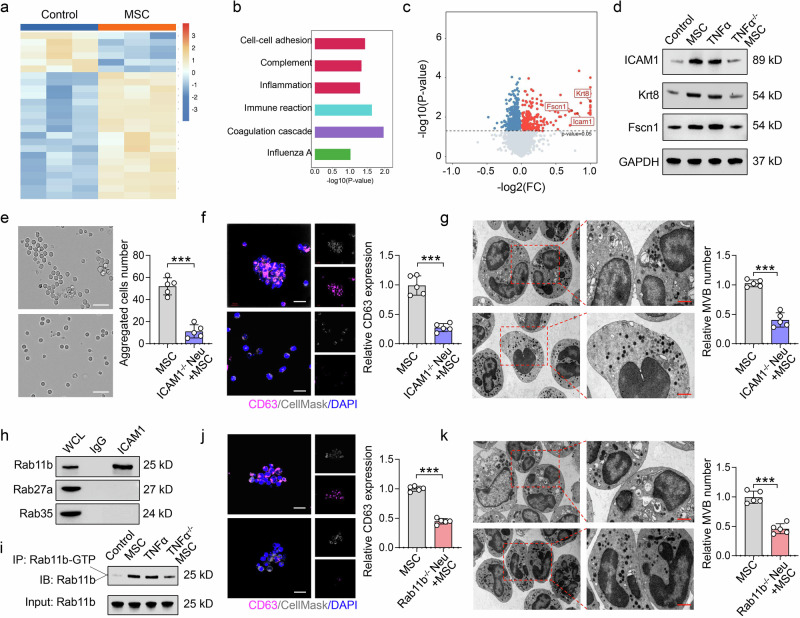
Neutrophil aggregation releases EVs via the ICAM1/Rab11b pathway. **a** Cluster heatmap analysis revealed 69 differentially expressed proteins between the neutrophil group and the MSC-treated neutrophil group. **b** KEGG pathway analysis revealed that the cell adhesion pathway was significantly different between the neutrophil group and the MSC supernatant-treated neutrophil group. **c** Volcano plot and **d** Western blotting results indicating that the levels of the cell–cell adhesion-related proteins ICAM1, Krt8, and Fscn1 were increased in the supernatant of MSC-treated neutrophils but not in the TNFα^−/−^ MSC supernatant; n = 3 per group. **e** The morphology of ICAM1 knockout neutrophils (ICAM1^−/−^ Neu) stimulated with MSC supernatant was analyzed *via* microscopy, n = 5. Scale bar = 20 μm. **f** CD63 expression in ICAM1^−/−^ Neu cells stimulated with MSC supernatant was detected *via* fluorescence microscopy, n = 5. Scale bar = 10 μm. **g** MVB production by ICAM1^−/−^ Neu cells stimulated with MSC supernatant was detected via transmission electron microscopy, n = 5. Scale bar = 1 μm. **h** CoIP assays revealed that ICAM1 interacted with Rab11b; WCL whole-cell lysate; n = 3. **i** Pulldown activation assays revealed that both the MSC supernatant and TNFα increased Rab11b-GTP expression, whereas the TNFα^−/−^ MSC supernatant did not, n = 3. **j**, **k** MSC supernatant induced neutrophil aggregation in Rab11b knockout mice (Rab11b^−/−^ Neu) but failed to activate CD63 expression and MVB formation in Rab11b^−/−^ Neu, n = 5. Scale bar = 20 μm (**j**), Scale bar = 1 μm (**k**). NS not significant; ***p < 0.001

To further elucidate the mechanism of MSC-induced neutrophil aggregation and subsequent EV production, we performed protein sequencing of MSC supernatant-treated neutrophils. A differential protein heatmap revealed that 63 proteins were upregulated and 6 proteins were downregulated in MSC supernatant-treated neutrophils relative to those in untreated neutrophils (Fig. 4a). The cell‒cell adhesion and junction pathway is necessary for cell clustering and cell aggregation.^33^ Pathway enrichment analysis revealed that cell–cell adhesion and junction pathway signaling was increased in MSC supernatant-treated neutrophils (Fig. 4b). Volcano plot results revealed that the expression levels of cell‒cell adhesion and junction molecules, including intercellular adhesion molecule 1 (ICAM1), keratin 8 (Krt8) and fascin 1 (Fscn1), were increased in MSC supernatant-treated neutrophils (Fig. 4c). The expression levels of ICAM1, Krt8 and Fscn1 were increased in neutrophils after MSC supernatant or TNFα stimulation, whereas the supernatant of TNFα^−/−^ MSCs failed to increase the expression of ICAM1, Krt8 and Fscn1 in neutrophils (Fig. 4d). Thus, these data indicate that MSC-derived TNFα induces neutrophil aggregation through cell‒cell adhesion and junctions in neutrophils.

To clarify which molecules are vital for MSC-induced neutrophil aggregation, we isolated ICAM1 knockout neutrophils (ICAM1^−/−^ Neu), Krt8 knockout neutrophils (Krt8^−/−^ Neu) and Fscn1 knockout neutrophils (Fscn1^−/−^ Neu) from the bone marrow of the respective genetic knockout mice. The results revealed that MSC supernatant failed to promote ICAM1^−/−^ neutrophil aggregation, CD63 expression and MVB formation (Fig. 4e–g), whereas MSC supernatant promoted Krt8^−/−^ and Fscn1^−/−^ neutrophil aggregation and CD63 expression (Supplementary Fig. 5a). Therefore, ICAM1 is an important downstream molecule for MSC supernatant-induced neutrophil aggregation.

Next, we explored the mechanism of EV production from neutrophil aggregation. The Rab family of small GTPases is essential for the endosomal pathway, which is closely associated with EV secretion.^34–36^ There are three main Rab-GTPases described in EV biogenesis and secretion: Rab11b, Rab27a, and Rab35.^36^ Immunoprecipitation‒mass spectrometry (IP‒MS) and coIP confirmed that ICAM1 can bind with Rab11b but not Rab27a or Rab35, suggesting that ICAM1 may exert its function through Rab11b (Fig. 4h, Supplementary Fig. 5b, Supplementary Table 1). We next utilized molecular docking and molecular simulation to further evaluate the function of TNFα in the interaction between ICAM1 and Rab11b. The results revealed that ICAM1 and Rab11b interact hydrophobically and that TNFα could enhance this interaction through the formation of hydrogen bonds (Supplementary Fig. 5c, d). In terms of molecular dynamics, the root-mean-square deviation (RMSD) was calculated to determine the conformational change in these regions throughout the simulation.^37^ The radius of gyration (Rg) was analyzed to determine the compactness and rigidity of the interacting proteins.^38^ The data revealed that TNFα promoted the interaction and stabilization of ICAM1 and Rab11b (Supplementary Fig. 5e, f). GTP-bound Rab is the active form of the Rab family. We found that both the MSC supernatant and TNFα, but not the supernatant of TNFα^−/−^ MSCs, facilitated the activation of Rab11b (Fig. 4i). These data confirmed that TNFα facilitates the interaction of ICAM1 and Rab11b and then promotes the activation of Rab11b.

Next, we isolated Rab11b knockout neutrophils (Rab11b^−/−^ Neu) from the bone marrow of Rab11b^−/−^ mice. MSC supernatant induced the aggregation of these neutrophils but failed to promote CD63 expression and MVB formation (Fig. 4j, k, Supplementary Fig. 5g). Collectively, these results indicate that MSC-induced neutrophil aggregation releases EVs through the ICAM1/Rab11b pathway.

### EV storm determines MSC efficacy in SLE mice and patients

**Fig. 5:**
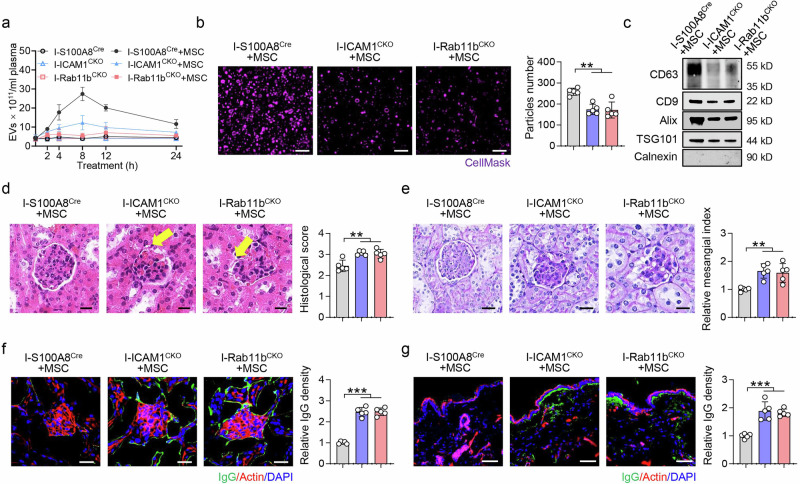
The ICAM1/Rab11b pathway is essential for EV storm and MSC-mediated therapy for SLE. **a** ZetaView assay showing the number of EVs in the blood of IMQ-treated ICAM1^CKO^ (I-ICAM1^CKO^) mice and IMQ-treated Rab11b^CKO^ (I-Rab11b^CKO^) mice at 2, 4, 8, 12, and 24 h postMSC transplantation; n = 3 per group. **b**, **c** The number of EVs and the expression of EV markers were lower at 8 h postMSC transplantation in the I-ICAM1^CKO^ and I-Rab11b^CKO^ mice than in the I-S100A8^Cre^ mice. n = 5, Scale bar = 1 μm (**b**), n = 3 (**c**). **d**, **e** H&E staining and PAS staining demonstrated that MSC transplantation failed to reduce the histological score and relative mesangial index of the kidney in I-ICAM1^CKO^ mice and I-Rab11b^CKO^ mice (n = 5). Scale bar = 20 μm. The arrow indicates a broken glomerular margin (**d**). Confocal microscopy confirmed that MSC transplantation failed to inhibit IgG deposition in the kidneys (**f**) and skin **g** of I-ICAM1^CKO^ mice and I-Rab11b^CKO^ SLE mice (n = 5). Scale bar = 20 μm. **p < 0.01; ***p < 0.001

To examine the molecular mechanism of MSC-induced EV storms in vivo, we generated S100A8^Cre^-ICAM1^fl/fl^ mice (ICAM1^CKO^ mice) and S100A8^Cre^-Rab11b^fl/fl^ mice (Rab11b^CKO^ mice). IMQ treatment subsequently induced the SLE phenotype in these mice (I-S100A8^Cre^; I-ICAM1^CKO^ mice and I-Rab11b^CKO^ mice). The SLE-associated markers (ANA, BUN and anti-dsDNA) and T-cell subset ratios (Th17 and Treg) did not significantly differ among the different groups (Supplementary Fig. 6a). MSC transplantation failed to trigger EV storms in the blood of I-ICAM1^CKO^ mice and I-Rab11b^CKO^ SLE mice (Fig. 5a–c). In addition, MSC treatment failed to induce neutrophil aggregation in the bone marrow of ICAM1^CKO^ mice (Supplementary Fig. 6b–d).

Next, we evaluated the relationship between EV storms and MSC efficacy in SLE mice. The efficacy of MSC transplantation was significantly lower in I-ICAM1^CKO^ mice and I-Rab11b^CKO^ mice than in I-S100A8^Cre^ control mice. In I-ICAM1^CKO^ mice and I-Rab11b^CKO^ mice, MSC transplantation failed to relieve kidney damage, alleviate IgG deposition, or normalize ANA, BUN and anti-double-stranded DNA (anti-dsDNA) concentrations (Fig. 5d–g and Supplementary Fig. 6e). Compared with I-S100A8^Cre^ mice, MSC treatment failed to decrease the Th17 cell ratio and increase the Treg cell ratio in the spleens of I-ICAM1^CKO^ and I-Rab11b^CKO^ mice (Supplementary Fig. 6f, g). Similarly, MSCs failed to decrease IL-17A levels and increase Foxp3 levels in the spleens of I-ICAM1^CKO^ and I-Rab11b^CKO^ mice (Supplementary Fig. 6h, i).

**Fig. 6:**
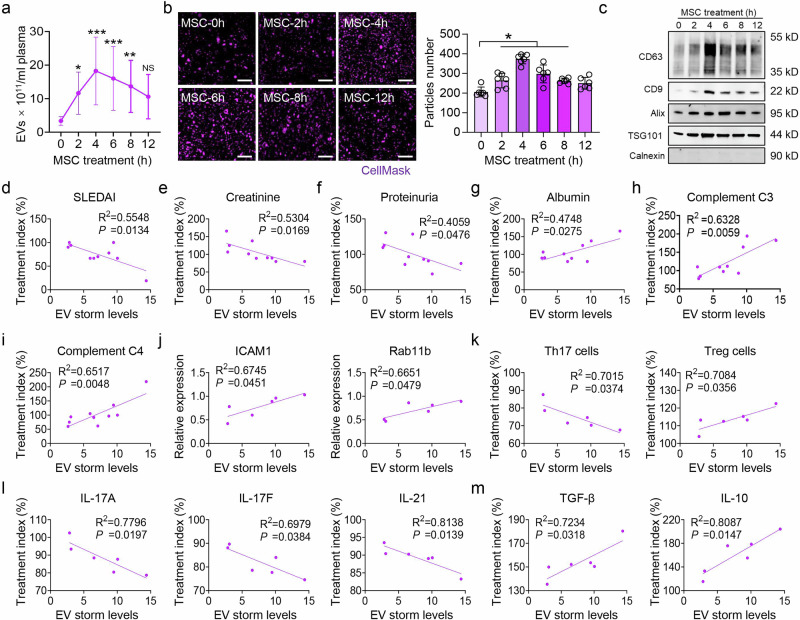
EV storm levels are related to the efficacy of MSC therapy in SLE patients. **a** A ZetaView assay revealed that the number of EVs in the blood significantly increased at 2, 4, 6, and 8 h postMSC transplantation in SLE patients (n = 15). **b** The number of EVs in the blood sample from patient #1 was detected by SIM; n = 5 per group. Scale bar = 1 μm. **c** The expression of EV-related markers in the blood of patients was examined by western blotting, n = 3. A higher EV storm was correlated with lower SLE disease activity index 2000 (SLEDAI; **d**), creatinine (**e**), and proteinuria (**f**) and higher levels of albumin (**g**), complement C3 (**h**), and complement C4 (**i**) (n = 10). The EV storm level is the ratio of (the highest number of EVs among 2, 4, 6, 8, and 12 h post-MSC transplantation)/(the number of EVs at 0 h post-MSC transplantation). The treatment index was calculated as (the value of the clinical index after 1 month of MSC transplantation)/(the value before MSC transplantation). **j** qRT‒PCR was used to analyze the relationship between EV storm levels and the expression of ICAM1 and Rab11b in the neutrophils of SLE patients (n = 6). **k** Flow cytometry analysis revealed that EV storm levels were negatively correlated with the treatment index of Th17 cells and positively correlated with the treatment index of Treg cells (n = 6). **l**, **m** ELISA revealed that EV storm levels were negatively associated with the treatment indices of the Th17 cell-related cytokines IL-17A, IL-17F, and IL-21 but positively associated with the treatment indices of the Treg cell-related cytokines IL-10 and TGF-β (n = 6). *p < 0.05

To verify the EV storm phenomenon in humans, 15 SLE patients who received MSC transplantation were recruited (2 males and 13 females; Supplementary Table 2). Intriguingly, the number of circulatory EVs was significantly increased at 2, 4, 6, and 8 h after MSC transplantation in SLE patients (Fig. 6a–c**;** Supplementary Table 3). These MSC-induced blood EVs strongly expressed neutrophil markers (CD66b, CD15, and CD16) and expressed low levels of CD68, CD3, B220, CD90, and CD31 (Supplementary Fig. 7a, b). Next, we collected 10 patients’ follow-up data to evaluate the relationship between EV storm rates and the efficacy of MSC transplantation for SLE patients (Supplementary Table 3). One month after MSC transplantation, patients with higher EV storm rates presented lower SLE disease activity index 2000 (SLEDAI) scores, serum creatinine levels, and proteinuria and higher levels of albumin, complement C3, and complement C4 (Fig. 6d–i). EV storm levels were not significantly correlated with red blood cell (RBC), white blood cell (WBC), platelet (PLT) or BUN levels (Supplementary Fig. 7c). In addition, we divided patients into responders and nonresponders according to the clinical SLE response index (SRI)-4 remission. The SRI-4 response is a landmark assessment defined as at least a 4-point reduction in the SLEDAI-2K score.^39^ On the basis of the SRI-4 response at the one-month follow-up, 4 patients were responders, and 6 patients were nonresponders. EV storm levels were greater in the blood of responders than in that of nonresponders **(**Supplementary Fig. 7d).

Then, we collected blood samples from 6 patients among the 10 follow-up patients for mechanistic analysis. The results revealed that patients with increased EV storm levels presented increased relative expression of ICAM1 and Rab11b in their neutrophils (Fig. 6j). Patients with higher EV storm rates also presented a lower Th17 cell ratio and a higher Treg cell ratio (Fig. 6k). Furthermore, flow cytometry analysis demonstrated that patients with higher EV storm levels expressed lower serum levels of the Th17 cell-related cytokines IL-17A, IL-17F and IL-21 (Fig. 6l) and higher serum levels of the Treg cell-related cytokines TGF-β and IL-10 (Fig. 6m). These results suggest that higher EV storm rates are associated with the efficacy of MSC therapy in SLE patients.

### EV storms modulate Th17/Treg balance *via* the LILRB4/STAT5/STAT3 axis

**Fig. 7:**
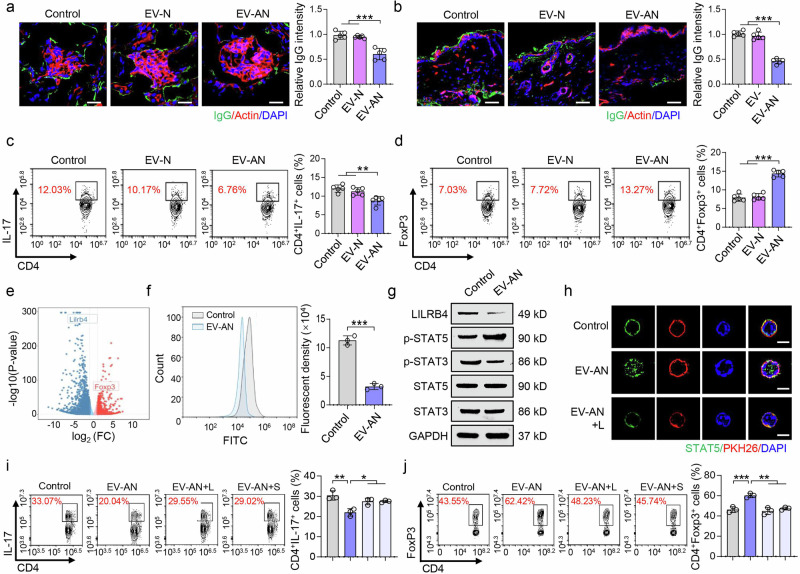
Aggregated neutrophil-derived EVs modulate Th17/Treg balance via the LILRB4/STAT5/STAT3 axis. **a**, **b** IgG deposition in the kidneys and skin of MRL/*lpr* mice was detected at 4 weeks after neutrophil-derived EV (EV-N) or aggregated neutrophil-derived EV (EV-AN) treatment; n = 5 per group. Scale bar = 20 μm. **c**, **d** The Th17/Treg cell ratio in the blood of MRL/*lpr* mice was analyzed by flow cytometry at 4 weeks after EV treatment (n = 5). **e** Volcano plot showing the gene expression pattern in EV-AN-treated CD4^+^ T cells. **f** Flow cytometry analysis confirmed that the expression of LILRB4 decreased in EV-AN-treated CD4^+^ T cells (n = 3). **g** Western blotting revealed that the expression of LILRB4 and p-STAT3 was decreased, whereas the expression of p-STAT5 was increased in EV-AN-treated CD4^+^ T cells (n = 3). **h** Immunofluorescence revealed that STAT5 was transferred to the cell nucleus in EV-AN-treated CD4^+^ T cells, whereas this effect was inhibited by the addition of LILRB4 (EV-AN + L), n = 3. Scale bar = 5 μm. **i**, **j** Flow cytometry was used to detect the ratios of Th17 (CD4^+^IL-17^+^) and Treg (CD4^+^Foxp3^+^) cells among the EV-AN, EV-AN + L and EV-AN + STAT5-IN-1 (EV-AN + S) groups; n = 3. *p < 0.05; **p < 0.01; ***p < 0.001

To elucidate the effect of EV storm on SLE, we separated mouse neutrophil-derived EVs (EV-Ns) and MSC-induced aggregated neutrophil-derived EVs (EV-ANs) and then injected them into MRL/*lpr* mice. Compared with EV-N, EV-AN inhibited IgG deposition in the kidney and skin at 4 weeks post-treatment (Fig. 7a, b). T-cell isotype disorders may contribute to the pathogenesis of SLE. Therefore, we further assessed the effects of EV-N and EV-AN on T-cell isotypes in the spleens of MRL/*lpr* mice (Supplementary Fig. 8a). Flow cytometry analysis revealed that EV-AN reduced the Th17 cell ratio and increased the Treg cell ratio at 4 weeks post-treatment, suggesting that EV-AN has a beneficial effect on modulating the Th17/Treg balance (Fig. 7c, d). However, the effects on the Th1 or Th2 cell ratio did not significantly differ between the EV-N and EV-AN treatment groups (Supplementary Fig. 8b).

To gain further insight into the cellular mechanisms through which EV storms modulate the Th17/Treg ratio, we analyzed the RNA sequencing data of mouse CD4^+^ T cells stimulated with EV-AN. EV-AN inhibited the expression of leukocyte immunoglobulin-like receptor B4 (LILRB4; Fig. 7e). LILRB4, an inhibitor receptor, is augmented in autoantibody-producing plasmablasts/plasma cells of SLE patients and mice.^24,40^ Flow cytometry and western blotting confirmed that EV-AN inhibited the expression of LILRB4 in CD4^+^ T cells (Fig. 7f, g).

STAT3 plays a crucial role in Th17 differentiation, whereas STAT5 is critical for Treg differentiation. Thus, the interaction between STAT3 and STAT5 is necessary for Th17/Treg balance and immune homeostasis. To examine mouse CD4^+^ T cells from WT mice, we found that EV-AN promoted the phosphorylation of STAT5 and inhibited the phosphorylation of STAT3 in vitro (Fig. 7g). In addition, EV-AN facilitated the nuclear translocation of STAT5 while restraining the nuclear translocation of STAT3 in CD4^+^ T cells (Fig. 7h, Supplementary Fig. 8c–e). However, the effects of EV-AN on STAT3 and STAT5 were impaired in the EV-AN + LILRB4 (EV-AN + L) group (Fig. 7h, Supplementary Fig. 8c–e). Furthermore, the STAT5 phospho-inhibitor also impaired the effects of EV-AN on STAT3 and STAT5 in the EV-AN + STAT5 phospho-inhibitor (EV-AN + STAT5-IN-1; EV-AN + S) group (Supplementary Fig. 8f). EV-AN promoted Treg cell differentiation and inhibited Th17 cell differentiation in vitro, while the addition of LILRB4 and STAT5-IN-1 impaired these effects (Fig. 7i, j). Compared with EV-AN + S-treated SLE mice, EV-AN + L-treated and EV-AN + S-treated SLE mice presented elevated histological scores, SLE indices (ANA, BUN, and anti-dsDNA), and Th17 cell ratios but reduced Treg cell ratios (Supplementary Fig. 8g–j). These data indicate that EV-AN balances the Th17/Treg cell ratio.

**Fig. 8:**
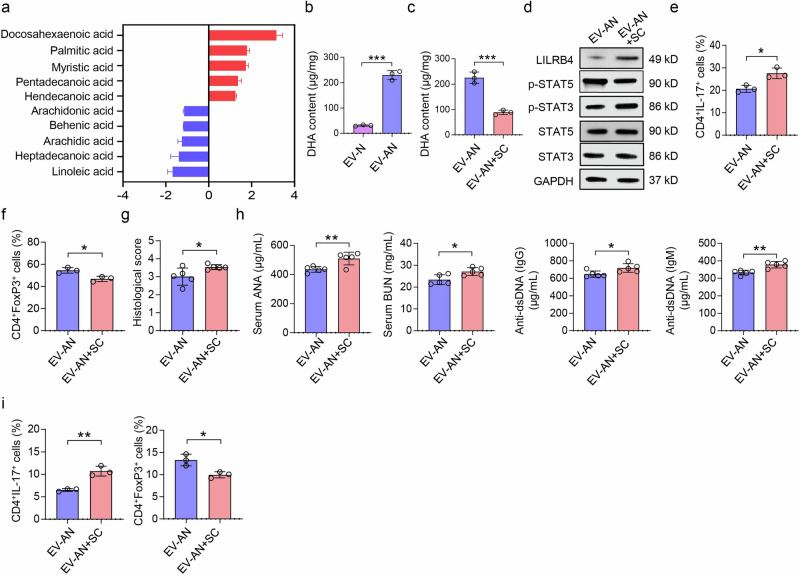
Aggregated neutrophil-derived EVs induce immune tolerance *via* DHA. **a** Quantitative analysis of long-chain free fatty acids in EVs derived from neutrophils (EV-Ns) and MSC-induced aggregated neutrophils (EV-ANs). **b**, **c** The concentration of DHA in the EV-AN and EV-AN + SC-26196 (AN + SC) groups; n = 3 per group. **d** The expression of LILRB4, p-STAT5 and p-STAT3 in the EV-AN and EV-AN + SC groups. **e**, **f** Flow cytometry was used to determine the ratios of Th17 (CD4^+^IL-17^+^) and Treg (CD4^+^Foxp3^+^) cells in vitro, n = 3. **g** Histological score index of the kidney in the EV-AN and EV-AN + SC groups, n = 5. **h** ELISA revealed that EV-AN + SC increased the levels of serum ANA, BUN, and anti-dsDNA, n = 5. **i** Flow cytometry was used to determine the ratios of Th17 and Treg cells in the spleen, n = 3. NS not significant; *p < 0.05; **p < 0.01; ***p < 0.01

To identify the EV-AN cargo responsible for balancing the Th17/Treg cell ratio, we performed metabolomic mass spectrometry on EV-Ns and EV-ANs. Docosahexaenoic acid (DHA) was significantly enriched in the EV-AN (Fig. 8a, b). Because DHA balances the Th17/Treg cell ratio,^41^ we used a DHA inhibitor (SC-26196) to pretreat MSC-induced aggregated neutrophils and then collected the EV-AN (EV-AN + SC). Compared with that in the EV-AN group, the DHA content in the EV-AN + SC group was significantly lower (Fig. 8c). Compared with EV-AN treatment, EV-AN + SC treatment increased the expression of LILRB4 and the phosphorylation of STAT3 while reducing the phosphorylation of STAT5 in CD4^+^ T cells in vitro (Fig. 8d). Compared with that of EV-AN group, the in vitro differentiation of Th17 cells was increased in EV-AN + SC group, whereas the in vitro differentiation of Treg cells was decreased (Fig. 8e, f). In addition, EV-AN + SC-treated SLE mice presented higher histological scores and higher levels of serum ANA, BUN and anti-dsDNA (Fig. 8g, h). Compared with EV-AN group, EV-AN + SC-treated SLE mice presented a greater Th17 cell ratio and lower Treg cell ratio (Fig. 8i). Together, these data support a critical role for DHA in the EV-AN-mediated balance of Th17/Treg cell ratio *via* the LILRB4/STAT5/STAT3 axis.

## Discussion

MSC transplantation has been shown to be safe and effective for a variety of immune diseases, such as SLE and graft-vs-host disease. However, some challenges prevent the broader adoption of MSC transplantation in the clinic. For example, the therapeutic mechanism is not fully understood, and the efficacy of MSC transplantation among patients is variable. Therefore, it is necessary to elucidate the therapeutic mechanism of MSCs in the disease treatment. Previous studies have focused on the modification and improvement of MSC quality, which is related to the therapeutic effect of MSCs in disease treatment.^42,43^ For example, priming MSCs with IFN-γ to upregulate IDO expression can be used to augment their immunosuppressive potency, which can improve the therapeutic effect of MSCs.^44^ Previous studies have shown that the therapeutic efficacy of MSCs is influenced by the host immune status, including the presence of inflammatory cytokines such as IFN-γ and the apoptotic MSC secretome.^6,7,45,46^ In this study, we show that an EV storm produced by recipients is necessary for MSC-mediated therapy for SLE, and the MSC-triggered EV storms in SLE mice are cell type-specific and dose-dependent.

Our data show that recipients react to donor MSCs and generate an EV storm in the early stage of MSC transplantation. This generation of an EV storm occurs 8 h after MSC transplantation in SLE mice and 2–6 h after MSC transplantation in SLE patients. In addition, A cytokine storm is characterized by excessive release of cytokines in the early stage of inflammation or pathogenic infection, which can cause organ injury, morbidity, and mortality.^47,48^ Previous study shows that EVs are important carriers and transmitters of cytokines, which exerts the message of cell interactions and tissue communication.^49^ Thus, whether altered cytokine levels accompany EV storms and whether EV storms is the carrier of cytokine storm should be clarified in future studies.

Previous studies have demonstrated that the priming of TNFα can improve the immunosuppressive effects of MSCs on immune cells, such as peripheral blood mononuclear cells (PBMCs) and macrophages.^50,51^ In addition, TNFα-exposed MSCs produce exosomes with enhanced immunoregulatory functions to promote M2 macrophage polarization.^52^ In this study, we demonstrated that MSC transplantation induces recipient neutrophil aggregation to generate an endogenous EV storm *via* TNFα. However, TNFα^−/−^ MSCs fail to induce this neutrophil aggregation and endogenous EV storm. Similarly, TNFα can promote the aggregate formation of hepatocytes.^31^ These results suggest that MSCs modulate neutrophils through TNFα-induced cell aggregation in the bone marrow of SLE mice. Besides, the homing of MSCs to the bone marrow might be required for neutrophil swarming, which is worthy of further investigation.

At local sites of tissue injury or infection, groups of individual neutrophils undergo phases of highly directed and coordinated movement, followed by cell accumulation and clustering.^53^ These coordinated processes are termed neutrophil aggregation (swarming), which constitutes an important mechanism of bodily self-protection. Previously, leukotriene B4 (LTB4) was identified as an essential initiator of neutrophil aggregation following laser-induced sterile inflammation in the dermis of the ear.^54^ Another study revealed that connexin-43 (Cx43) is critical for the initiation of swarming and promotes wound defense against opportunistic bacterial invasion.^55^ In this study, we report that ICAM1 mediates neutrophil aggregation after MSC transplantation. In addition, previous studies have indicated that the migration of neutrophils from the bone marrow to the infection site is the first step of neutrophil aggregation.^15^ In contrast, our results show that neutrophil aggregation induced by MSCs can generate an endogenous EV storm to exert immunoregulatory effects without migrating from the bone marrow. These results demonstrate that MSC-induced neutrophil aggregation represents a previously unknown pattern of swarming.

The expression of LILRB4, an inhibitory receptor, is increased in the plasma cells of SLE patients and mice.^24,40^ The “paradoxical” upregulation of LILRB4 in pathogenic plasma cells means that LILRB4 has multiple roles in the development and progression of SLE.^24^ LILRB4 can support tumor cell infiltration into tissues and suppress T-cell activity *via* the LILRB4/SHP-2/uPAR/Arginase-1 signaling axis in acute myeloid leukemia (AML) cells.^22^ However, the function of LILRB4 in CD4^+^ T-cell polarization is largely unknown. In this study, we collected EVs from the blood of SLE mice and patients after MSC transplantation. These data revealed that EVs can promote Treg differentiation and inhibit Th17 cell differentiation *via* the LILRB4/STAT5/STAT3 axis, suggesting that LILRB4 is a vital modulator of Th17/Treg balance. As an immune checkpoint, LILRB4 is an emerging target for the treatment of autoimmune diseases and cancer.^22,56^ We report here that the endogenous EV storm from MSC-treated SLE patients inhibits the expression of LILRB4, suggesting that the EV storm is an important inhibitor for excessive immune response, and EV storm may be leveraged in the treatment of other autoimmune diseases.

This study revealed that MSC transplantation can induce recipient neutrophil aggregation in the bone marrow, which generates an endogenous EV storm in the circulation of SLE mice and patients *via* the TNF/ICAM1/Rab11b pathway. EV storm exerts the function of rebalancing the Th17/Treg ratio in SLE mice *via* the DHA/LILRB4/STAT5/STAT3 axis. The inhibition of DHA content in EV storm could impair the therapeutic effect of EV storm in SLE mice. Interestingly, the level of the EV storm is positively correlated with the efficacy of MSC transplantation in SLE patients, suggesting that the EV storm can be used as an early predictor of the effectiveness of MSCs for SLE. These findings suggest that elements of the recipient response affect the efficacy of MSC transplantation and reveal a new therapeutic mechanism underlying MSC therapy.

In this study, we recruited 10 SLE patients to verify the relationship between EV storm and clinical response after MSC infusion. This observation is a preliminary test, and more clinical data are needed to validate this conclusion in future studies. In addition, this study proposes that neutrophil-derived EV storms can balance the Th17/Treg cell ratio *via* the DHA/LILRB4/STAT5/STAT3 axis. However, the potential contributions of nonneutrophil EVs require the further investigation. Additionally, it is possible that blood-derived neutrophils may also produce EV storms in the MSC treatment on SLE.

## Methods

### Animals

B6. MRL-Faslpr/J mice (MRL/*lpr* mice, JAX, #000482), B6.129S-Tnftm1Gkl/J mice (TNFα^−/−^ mice, JAX, #005540) and C57BL/6-Tg-CAG-EGFP-1Osb/J mice (GFP mice, JAX, #003291) were purchased from the Jackson Laboratory. C57BL/6JGpt-*ICAM1*^*em1Cd6804*^/Gpt (ICAM1-KO, Strain No. T002641), C57BL/6JGpt-*Krt8*^*em1Cd1931*^/Gpt (Krt8-KO, Strain No. T011851), and C57BL/6JGpt-*Fscn1*^*em3Cd19868in4*^/Gpt mice (Fscn1-KO, Strain No. T028851) were purchased from GemPharmatech (Nanjing, China). S100A8^Cre^-ICAM1^fl/fl^ mice were obtained by crossing C57BL/6JGpt-*H11*^*em1Cin(hS100A8-iCre)*^/Gpt (S100A8^Cre^, Strain No. T005636) with C57BL/6JGpt-*ICAM1*^*em1Cflox*^/Gpt mice (ICAM1-flox, Strain No. T052194).^57^ S100A8^Cre^-Rab11b^fl/fl^ mice were obtained by crossing S100A8^Cre^ with C57BL/6JGpt-*Rab11b*^*em1Cflox*^/Gpt mice (Rab11b-flox, Strain No. T019959).

### Antibodies and reagents

All the antibodies, cytokines, kits, and other resources used in this study are listed in the Key Resources Table.

### SLE models

IMQ was used to induce lupus-like phenotypes in WT mice as described previously.^58^ IMQ (5%) was applied topically to the inner ear three times a week for 8 weeks. In this study, 16-week-old IMQ-induced mice and MRL/*lpr* mice were used as SLE mouse models for subsequent MSC transplantation.

### Isolation of bone marrow MSCs

Bone marrow MSCs were harvested from the femurs of 6–8-week-old C57BL/6 or TNFα^−/−^ mice. The marrow was flushed with PBS containing 5% fetal bovine serum (FBS; Gibco, NY, USA), and the resulting cell suspension was passed through a 70 μm cell strainer (BD Biosciences, CA, USA) to obtain a single-cell suspension. The cells were plated and incubated at 37 °C with 5% CO₂ for 48 h. Nonadherent cells were removed by washing with PBS. The remaining adherent cells were cultured in alpha-MEM (Gibco) supplemented with 10% FBS, 2 mM L-glutamine, 50 μM 2-mercaptoethanol, 100 U/ml penicillin, and 100 μg/mL streptomycin for 10 days to allow for MSC expansion.

### The transplantation of cells and extracellular vesicles

Human umbilical cord mesenchymal stromal cells (MSCs) and bone marrow-derived MSCs from TNFα^−/−^ mice (TNFα^−/−^ MSCs) at passages 2–5 were used in this study. For most in vivo and in vitro mouse experiments, we used bone marrow-derived MSCs isolated from C57BL/6 and TNFα^−/−^ mice. For the human SLE trial, we used human umbilical cord-derived MSCs. For MSC transplantation, 1 × 10^6^ MSCs were administered to IMQ-induced and MRL/*lpr* mice *via* the tail vein. In addition, a lower MSC concentration (MSC-L; 0.1 × 10^6^) and a higher MSC concentration (MSC-H; 2 × 10^6^) were used to evaluate the effects of the MSC concentration on the degree of EV storm. After 4 weeks, all the treated mice were sacrificed for further research. Neutrophils and T cells were isolated from C57BL/6 mice to serve as representative allogeneic non-MSC cell types. The same number of neutrophils and T cells was injected into the SLE mice. For EV transplantation, 1 × 10¹⁰ EVs were administered intravenously to SLE mice.^59^ For EV blockade, GW4869 was administered to recipient mice 1 h before MSC infusion via an intraperitoneal injection at 2.5 mg/kg.^60^

### EV isolation from blood, tissue and culture supernatants

For blood EV isolation,^25,61^ plasma collected at a specific time point was centrifuged at 3200 *×* *g* for 20 min at 4 °C. Then, the plasma was sequentially centrifuged at 500 × *g* for 10 min, 3000 *×* *g* for 20 min and then at 12,000 *×* *g* for 20 min. The EVs were pelleted via ultracentrifugation at 100,000 *×* *g* for 70 min and washed once with PBS. For tissue EV isolation,^62^ the mice were euthanized via isoflurane inhalation, and the hearts were perfused with ice-cold PBS for 5 min. The tissues were extracted, chopped, and mixed with scalpel blades for 30 s on ice. Then, the suspension was passed through a 100-μm cell strainer and sequentially centrifuged at 500 *×* *g* for 10 min, 3000 *×* *g* for 20 min and then at 12,000 *×* *g* for 20 min. EVs were pelleted by ultracentrifugation at 100,000 *×* *g* for 70 min and washed with PBS. For EV isolation from culture supernatants, the supernatants of MSC-activated neutrophil aggregates were collected. The supernatants were sequentially centrifuged at 500 *×* *g* for 10 min, 3000 *×* *g* for 20 min and then at 12,000 *×* *g* for 20 min. EVs were pelleted by ultracentrifugation at 100,000 *×* *g* for 70 min and washed with PBS. The source, application and volume of EVs are summarized in Supplementary Table 4.

### EV detection

The EV concentration and size distribution were determined via ZetaView and nanoflow cytometry (nFCM).^63^ For the ZetaView assay, three cycles were performed by scanning 11 positions each and capturing 60 frames per position. After capture, the videos were analyzed via ZetaView software. For the nFCM assay, two single photon-counting avalanche photodiodes (APDs) were used for the simultaneous detection of side scatter (SSC) and fluorescence of individual particles. In addition, the expression of EV markers was detected via Western blotting and Elyra 7 Lattice SIM (Zeiss, Jena, Germany). For CellMask staining, we incubated EVs with the membrane dye CellMask DeepRed (1:4000) for 30 min at 37 °C and then washed the EVs with filtered PBS for SIM imaging.

### T-cell subset analysis

Naïve CD4⁺ T cells were isolated from the spleens of C57BL/6 mice via a naïve CD4⁺ T-cell isolation kit (BD Biosciences) following the manufacturer’s protocol. Purified cells were cultured in RPMI 1640 medium. The cells were activated with plate-bound anti-CD3 and anti-CD28 antibodies (2 μg/ml each). For Th17 cell differentiation, IL-1β (10 ng/ml), IL-6 (20 ng/ml), IL-23 (10 ng/ml), TGFβ (1 ng/ml), anti-mouse IL-4 (5 mg/ml) and anti-mouse IFN-γ (10 mg/ml) were added to the cell cultures. For Treg differentiation, TGF-β1 (10 ng/ml) and IL-2 (100 U/ml) were added. STAT5-IN-1 (10 μM) was used to pretreat EV-AN-induced T cells for 1 h.

### In vivo imaging system (IVIS) analysis

IVIS was used to analyze the distribution of EVs in vivo. Twelve hours before MSC transplantation, the mice were treated with an anti-CD63 antibody. Then, the mice were sacrificed, and the organs were isolated for IVIS analysis. The fluorescence intensity was used to evaluate the levels of EVs in different organs.

### SLE patient analysis

Fifteen SLE patients who underwent MSC transplantation were recruited for this study. All patients fulfilled the American College of Rheumatology criteria for SLE. Briefly, MSCs were administered *via* intravenous infusion (2 × 10^6^/kg body weight).^64^ The patients’ plasma was collected at 0, 2, 4, 6, 8, and 12 h postMSC transplantation, after which the EVs were sequentially centrifuged. The number of EVs was analyzed via ZetaView. EV storm levels are calculated as follows: (the highest number of EVs at 2, 4, 6, 8, and 12 h post-MSC transplantation)/(the number of EVs at 0 h post-MSC transplantation).

To evaluate the relationship between EV storm rates and the efficacy of MSC therapy for SLE, the clinical indices of SLE patients 1 month after MSC transplantation were collected. The SLE Disease Activity Index 2000 (SLEDAI) score, which includes descriptors of mucosal ulcers, rash, and alopecia, was used to assess SLE patients’ disease activity.^65^ The treatment index was calculated as (the value of the clinical index after 1 month of MSC transplantation)/(the value before MSC transplantation). In addition, the plasma of patients 1 month after MSC transplantation was collected for ELISA. Peripheral blood mononuclear cells (PBMCs) were isolated for analysis of the Th17/Treg cell ratio.

### Tissue-clearing technology

The mice were anesthetized, and cardiac perfusion was performed with ice-cold 1× PBS followed by ice-cold 4% PFA solution. The femurs were harvested and incubated in 4% PFA at 4 °C with shaking overnight and then washed with 1× PBS for at least 2 h three times at room temperature. The fixed femurs were decalcified using Nuohai Decalcifying Reagent at room temperature until the bones became soft. A Tissue Clearing Kit was used to clarify the samples. Briefly, samples were immersed individually in 4 ml of Solution A for 6‒7 days (the solution was replenished twice a day) with gentle shaking at 37 °C and then washed with 1× PBS for at least 6 h (with refreshing every hour) with gentle shaking at room temperature. The cleared samples were subsequently subjected to immunostaining by sequentially immersing them in primary and secondary antibody solutions, each for 7 days at 37 °C with gentle shaking and with 1× PBS washes in between. Finally, the samples underwent refractive index (RI) matching, and 3D fluorescence imaging was conducted with a Nuohai LS 18 Tiling Light Sheet Microscope. The 3D images were further analyzed and processed *via* Imaris.

### Tissue Microarray Analysis (TMA)

The TMA module of HALO^®^ v3.6.4134 image analysis software (Indica Labs) was used to analyze immunohistochemical images of mouse femoral bone marrow. Specifically, complete longitudinal images of the mouse femurs were segmented into microarrays, Ly6G-positive expression was analyzed in each microregion, and the proportion of positive cells was assessed through high-throughput analysis to indirectly reflect the degree of neutrophil aggregation.

### Neutrophils stimulated with the supernatant of MSCs

To analyze the effects of MSCs on neutrophils, the supernatant of the MSCs was collected and sequentially centrifuged to remove the EVs. Then, the neutrophils were stimulated with the MSC supernatant. The proliferation, apoptosis, and aggregation of neutrophils were analyzed. MSCs were pretreated with SC-26196 (100 nM) for 1 h to induce neutrophil aggregation.

### LC‒MS/MS analysis

Mass spectrometry was performed on an Orbitrap Q Exactive HF-X mass spectrometer coupled with an UltiMate 3000 LC system (Thermo Fisher, Waltham, MA, USA). The samples were reconstituted in formic acid and transferred into a precolumn, after which the precolumn was connected to a microcapillary analytical column. HCD MS/MS spectra were recorded in data-dependent mode *via* the top-20 method. The raw data files were searched against the *Mus musculus* UniProt canonical database with pFind 3.0 studio (http://pfind.ict.ac.cn/software/pFind3/index.html).

### Western blotting

Coimmunoprecipitation was performed via a Pierce MS-compatible Magnetic IP Kit. Briefly, the cell lysates were incubated with the antibody at 4 °C overnight, followed by incubation with protein A/G magnetic beads for 2 h. After washing five times, the precipitates were eluted for immunoblotting and mass spectrometric detection. Western blotting was performed as previously described.^66,67^ Total protein was extracted with a total protein extraction kit (Thermo Fisher) following the manufacturer’s protocol. For western blotting, 20 mg of protein was subjected to electrophoresis and transferred to PVDF membranes (Millipore, Billerica, MA, USA). After blocking for 1 h, the membranes were incubated with the corresponding primary antibody at 4 °C overnight and then incubated with secondary antibodies for 1 h. The membranes were visualized via SuperSignal West Pico Chemiluminescent Substrate (Thermo Fisher) and scanned via a ChemiDoc MP imaging system (Bio-Rad, CA, USA).

### Quantitative PCR analysis

Total RNA was extracted *via* TRIzol reagent (Life Technologies, MD, USA), and cDNA was synthesized *via* a reverse transcriptase M-MLV Kit (TaKaRa, Tokyo, Japan). Gene expression levels were quantified *via* qRT‒PCR with a SYBR Green kit (Roche, Basel, Switzerland) with gene-specific primers (Supplementary Table 5).

### Rab11b activity assay

Rab11b activity was determined via a Rab activity assay kit (NewEast Biosciences, King of Prussia, PA) according to the manufacturer’s protocol. Briefly, cell lysates containing equal amounts of total proteins were incubated with a mouse monoclonal antibody specifically recognizing guanosine triphosphate (GTP)-bound Rab11b. Bound active Rab11b was pulled down with protein A/G agarose and detected with a rabbit polyclonal anti-Rab11b antibody.

### Flow cytometry

For intracellular cytokine staining, the cells were stimulated with phorbol 12-myristate 13-acetate (PMA, 50 ng/mL), ionomycin (1 mg/mL) and monensin (GolgiStop; 1 mg/mL) for 4 h. After surface marker staining, the cells were permeabilized with a FoxP3/transcription factor staining kit (Thermo Fisher, CA, USA) and intracellularly stained with the corresponding antibodies. The labeled antibodies included APC-conjugated anti-mouse CD4, FITC-conjugated anti-mouse IFN-γ, PE-conjugated anti-mouse IL-4, PE-conjugated anti-mouse IL-17, and PE-conjugated anti-mouse Foxp3. For the apoptotic rate assay, the cells were stained with FITC-conjugated anti-Annexin V and PE-Cy7-conjugated anti-7AAD for 15 min at room temperature. The cells were subsequently analyzed *via* flow cytometry (NovoCyte, Agilent Technologies, CA, USA).

### Histology

Renal tissues from different groups were fixed in 10% buffered formalin. Paraffin sections were prepared and stained with H&E and PAS for renal damage evaluation. After H&E staining, the pathological score was graded from 0 to 4: 0, normal; 1, a small increase in the number of cells in the glomerular mesangium; 2, a greater number of cells in the mesangium; 3, glomerular lobular formation and a thickened basement membrane; and 4, glomerular crescent formation, sclerosis, tubular atrophy, and casts. The score for each animal was calculated by dividing the total score by the number of glomeruli observed.^66^ PAS staining was used to evaluate mesangial expansion *via* the mesangial index (MI).^66^ The MI value was calculated as the PAS^+^ area (pixels) of the glomerulus/total area (pixels) of the glomerulus.

For IgG deposition evaluation, frozen renal and skin tissue sections were prepared and stained with Alexa Fluor 488-conjugated anti-mouse IgG. For tissue apoptotic rate analysis, frozen spleen tissue sections from different groups were prepared and stained with a DeadEnd Fluorometric TUNEL kit (Promega).

### Statistical analysis

Normal distribution was assessed via the Shapiro‒Wilk test with sample sizes greater than 6. Comparisons between two groups were analyzed *via* independent unpaired two-tailed Student’s *t* tests for normally distributed data. Comparisons between more than two groups were analyzed via one-way ANOVA with Tukey’s test. *P* values less than 0.05 were considered statistically significant. The animals were randomly allocated via a random number generator. Blinding was implemented during data analysis. Sample sizes were determined on the basis of power analysis and previous literature.

### Study approval

Blood samples from SLE patients were obtained from the Medical Examination Centre of Nanjing Drum Tower Hospital. Informed consent was obtained from all the participants. This study was approved by the Ethics Committee at Nanjing Drum Tower Hospital and registered at http://ClinicalTrials.gov (identifier: NCT01741857). All animal experiments were approved by the Animal Ethical and Welfare Committee of Sun Yat-Sen University (SYSU-IACUC-2024-002237).

## Supplementary information

Table S1. IP-MS

Supplementary_Materials

Schematic diagram

wb raw figures

## Data Availability

The data that support the findings of this study are available in Figshare (10.6084/m9.figshare.30138400.v1). The Supporting Data Values file is provided as supplemental material.
