# Supplementary material for: Mesenchymal stromal cells induce neutrophil aggregation and extracellular vesicle storms for systemic lupus erythematosus
Source: Signal Transduct Target Ther. 2025 Oct 13;10:344. doi: 10.1038/s41392-025-02442-1 (PMC12518853; doi:10.1038/s41392-025-02442-1)
Supplement: Supplementary file 2 — Supplementary_Materials [file 41392_2025_2442_MOESM2_ESM.docx]

Supplementary Materials for

Mesenchymal stromal cells induce neutrophil aggregation to generate extracellular vesicle storms for systemic lupus erythematosus therapy

Qianmin Ou, Luhan Niu, Dandan Wang, Genhong Yao, Qianhui Ren, Zhengshi Li, Xueli Mao, Wei Teng, Zetao Chen, Andy Peng Xiang, Songtao Shi, Lingyun Sun

Correspondence to: Songtao Shi (shisongtao@mail.sysu.edu.cn), Lingyun Sun (lingyunsun@nju.edu.cn)

**This PDF file includes:**

Figures. S1 to S8

Tables S2 to S5

Key resources table

Fig. S1


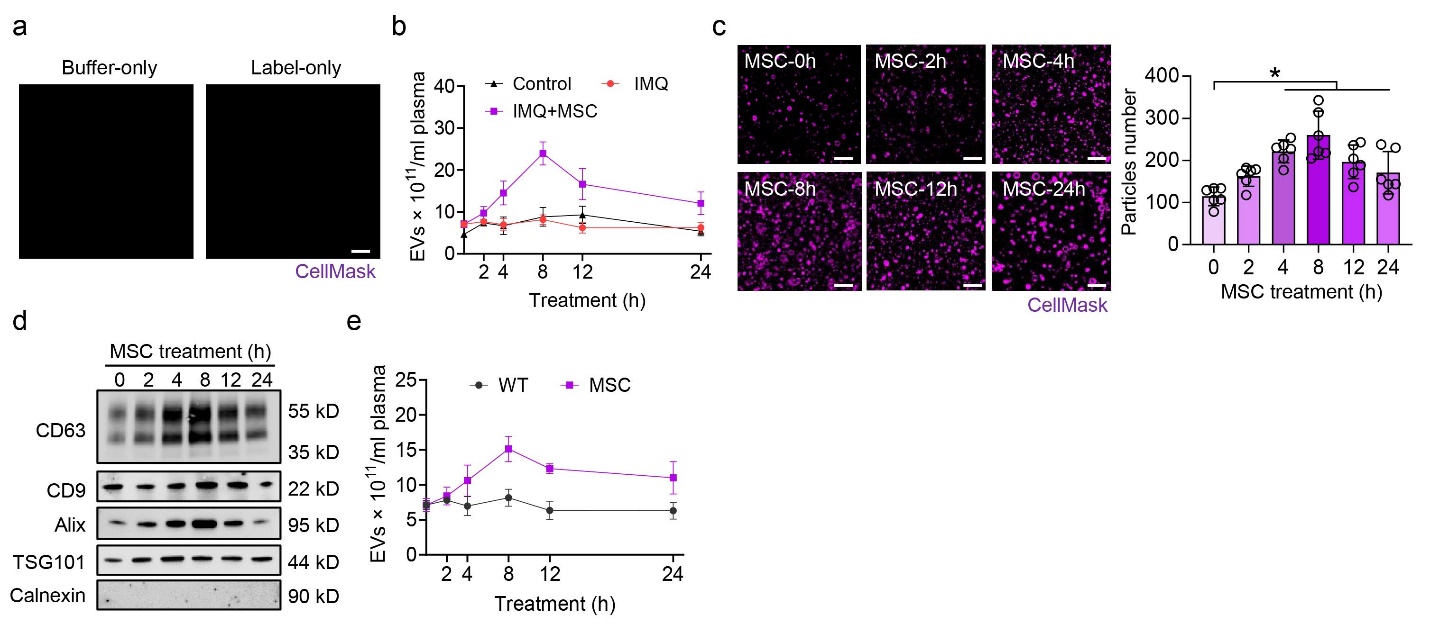


Fig. S1 MSC transplantation triggers EV storms in imiquimod-induced SLE mice and wild-type mice.

(**a**) Buffer-only and label-only controls for SIM. Scale bar = 1 μm. (**b**) ZetaView assay results indicating that MSC transplantation promoted EV production in the blood of imiquimod (IMQ)-induced SLE mice at 2, 4, 8, 12 and 24 hours postMSC transplantation; n = 3 per group. (**c**) SIM and (**d**) western blotting demonstrated that the number of EVs in the blood and the expression of EV markers were increased in IMQ-induced SLE mice at 4, 8, 12 and 24 hours after MSC transplantation. n = 5; scale bar = 1 μm (**c**), n = 3 (**d**). (**e**) A ZetaView assay revealed that MSC transplantation increased the number of EVs in the blood of wild-type mice at 4, 8, 12 and 24 hours postMSC transplantation (n = 3). **p* < 0.05.

Fig. S2


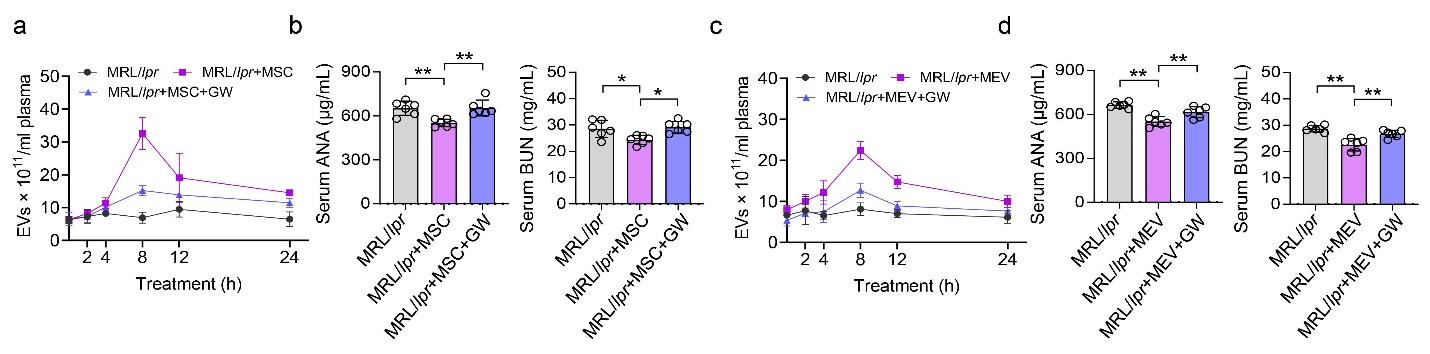


Fig. S2 EV storms are necessary for SLE treatment.

(**a**) ZetaView assay was used to detect the blood EVs of MRL/*lpr* mice at 4, 8, 12 and 24 hours after treatment with MSCs and GW. GW: GW4869; n = 3 per group. (**b**) ELISA was used to analyze the levels of serum ANA and BUN in MRL/*lpr* mice treated with MSCs or GW4869, n = 6. (**c**) A ZetaView assay was used to detect the blood EVs of MRL/*lpr* mice at 4, 8, 12 and 24 hours after treatment with MEV or GW4869, n = 3. (**d**) ELISA was used to analyze the levels of serum ANA and BUN in MRL/*lpr* mice treated with MEV or GW4869, n = 6. **p* < 0.05, ***p* < 0.01.

Fig. S3


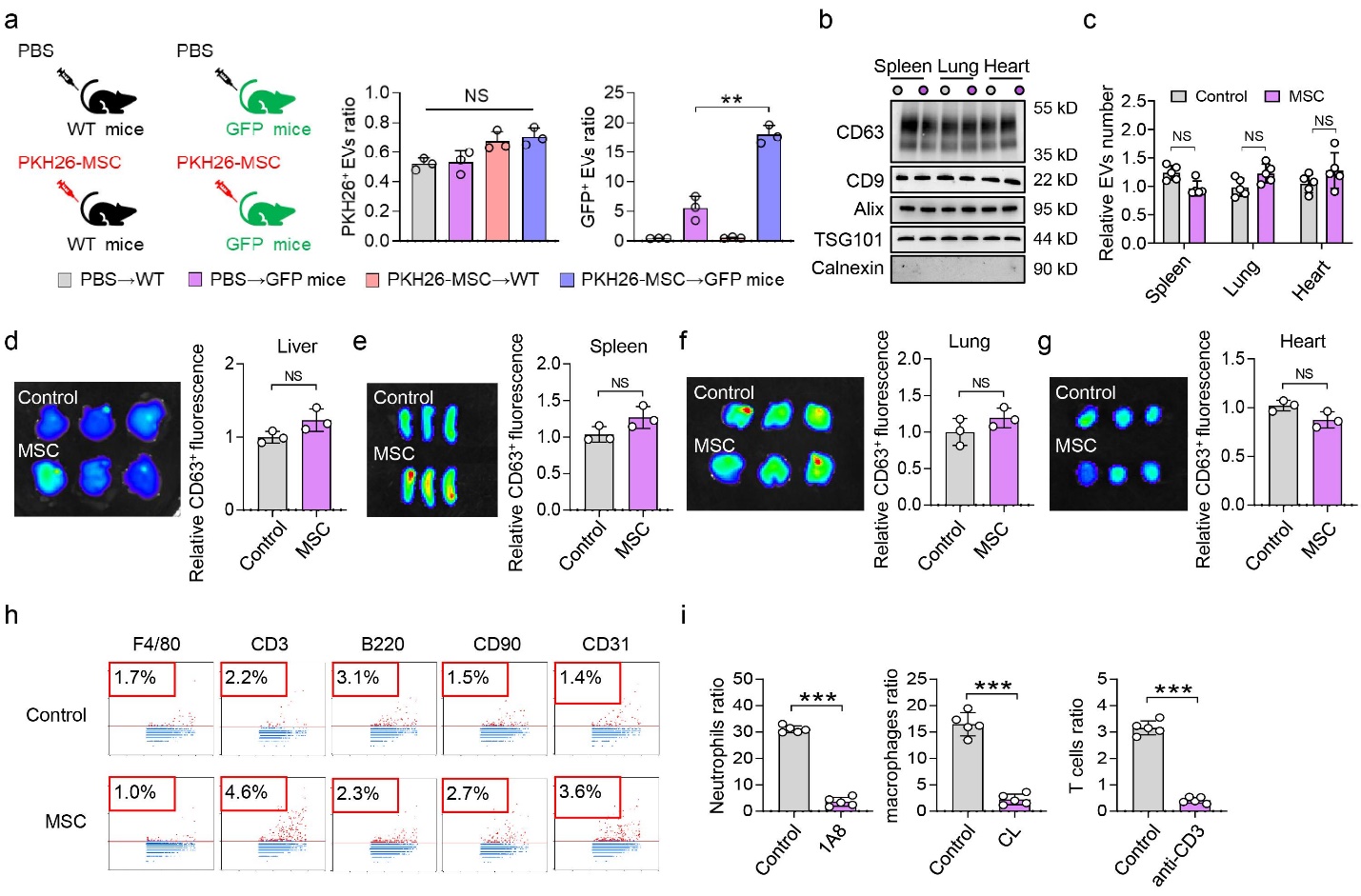


Fig. S3 EV storms mainly originate from recipient neutrophils.

(**a**) Schematic diagram of PKH26-labeled MSC injection into WT mice and GFP-expressing mice. nFCM analysis was used to determine the ratio of PKH26^+^ EVs to GFP^+^ EVs in the blood of the mice, n = 3 per group. (**b**) Western blotting and (**c**) ZetaView analysis revealed that the levels of EVs in the spleen, lung and heart did not significantly change at 8 hours postMSC transplantation (n = 3 (**b**), n = 5 (**c**)). (**d-g**) CD63^+^ fluorescence in the liver (**d**), spleen (**e**), lung (**f**) and heart (**g**) at 8 hours postMSC transplantation; n = 3. (**h**) nFCM results showing the ratios of F4/80, CD3, B220, CD90 and CD31 in the blood EVs of MSC-treated MRL/*lpr* mice, n = 3. (**i**) Flow cytometry was used to analyze the ratios of neutrophils, macrophages and CD4^+^ T cells in the bone marrow of MRL/*lpr* mice after treatment with 1A8, CL, or anti-CD3, respectively (n = 5). NS, not significant; ***p* < 0.01, ****p* < 0.001.

Fig. S4


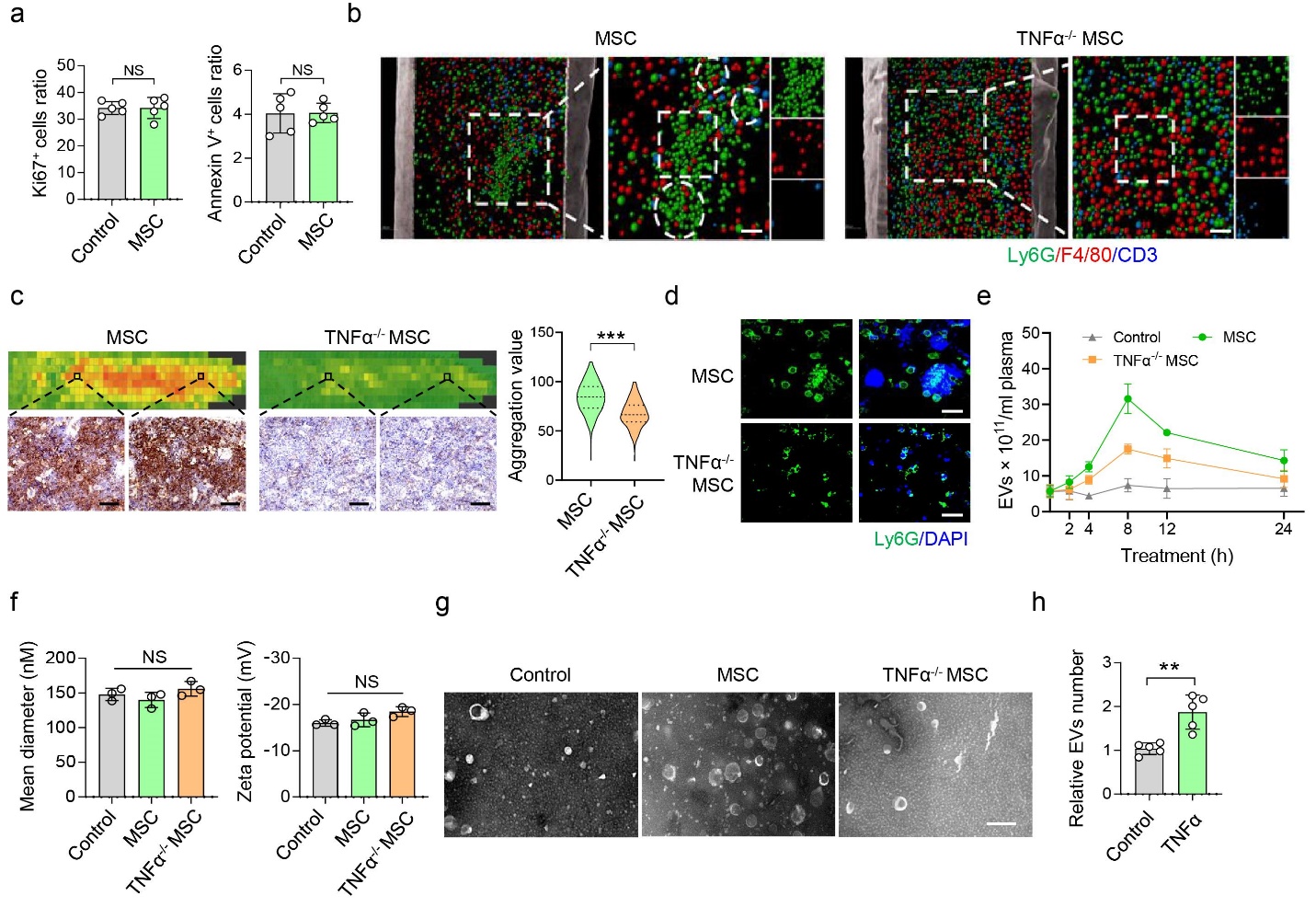


Fig. S4 TNFα^-/-^ MSCs fail to induce an EV storm *in vivo* and *in vitro*.

(**a**) Flow cytometry analysis revealed that the proliferation and apoptosis of bone marrow-derived neutrophils did not significantly differ between the two groups (n = 5 per group). (**b-d**) Tissue transparency experiments (**b**), HALO proximity analysis (**c**) and bone marrow smears (**d**) indicated that TNFα^-/-^ MSCs failed to induce significant neutrophil aggregation in the bone marrow of MRL/*lpr* mice (n = 3). Scale bar = 200 μm (**b**), Scale bar = 40 μm (**c**), Scale bar = 20 μm (**d**). (**e**) ZetaView assay results showing that TNFα^-/-^ MSCs failed to promote EV production in the blood of MRL/*lpr* mice, n = 3. (**f**) ZetaView assay showing the mean diameter and zeta potential of blood EVs isolated from MRL/*lpr* mice treated with MSCs or TNFα^-/-^ MSCs, n = 3. (**g**) TEM images showing the typical cup-shaped morphology of EVs from each group; scale bar = 200 nm. (**h**) ZetaView assay was used to detect the relative number of EVs in TNFα-treated neutrophils *in vitro*, n = 5. NS, not significant; ***p* < 0.01.

Fig. S5


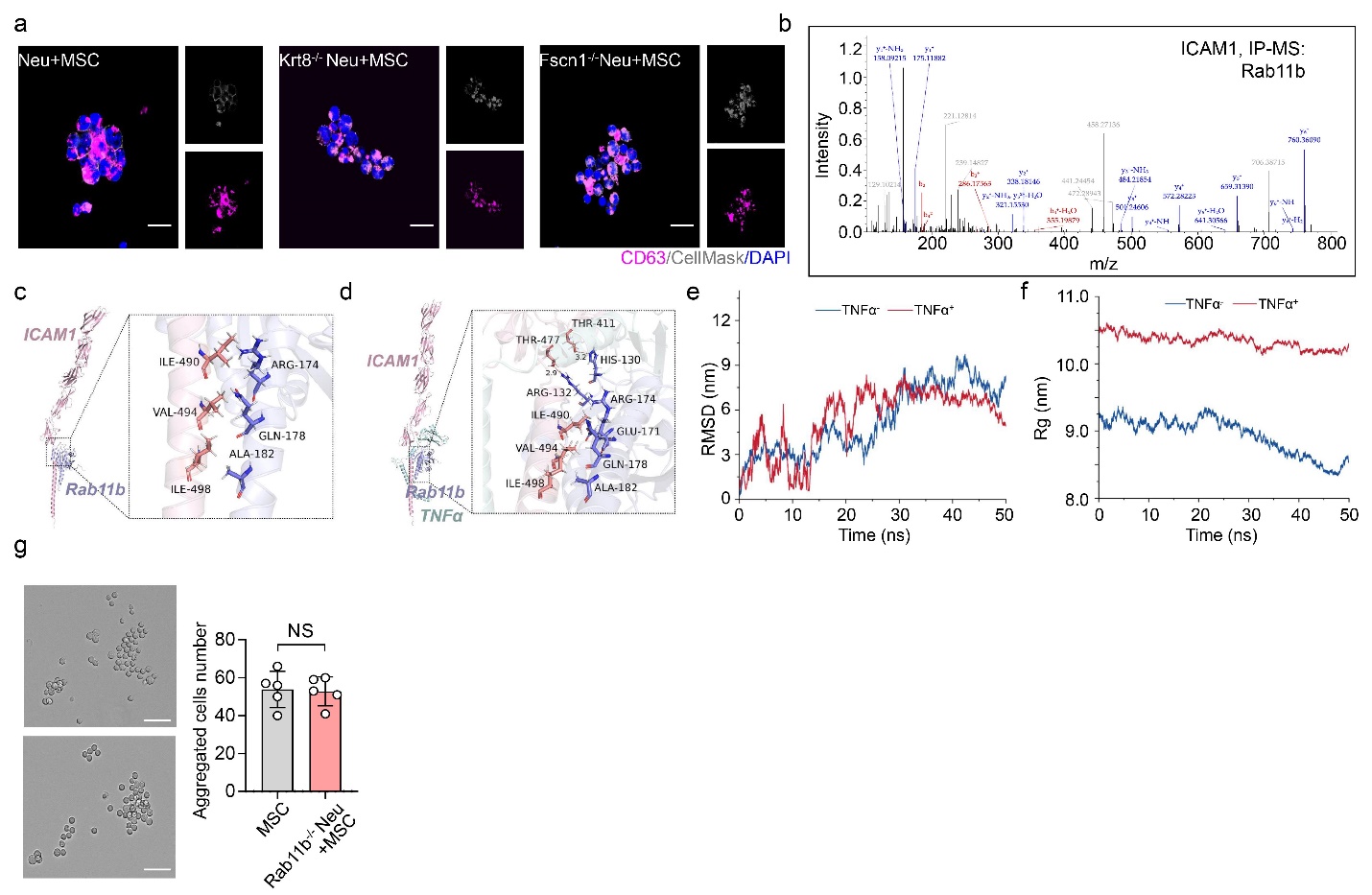


Fig. S5 The ICAM1/Rab11b pathway is required for MSC-triggered EV storm.

(**a**) Immunofluorescence revealed that MSC transplantation induced cell aggregation and CD63 expression in Krt8 knockout neutrophils (Krt8^-/-^ Neu) and Fscn1 knockout neutrophils (Fscn1^-/-^ Neu), n = 5 per group. Scale bar = 10 μm. (**b**) IP-MS showing the interaction between ICAM1 and Rab11b. (**c, d**) Molecular docking demonstrated that ICAM1 and Rab11b have hydrophobic interactions (**c**) and that TNFα enhances these interactions through the formation of hydrogen bonds (**d**). (**e,** **f**) TNFα treatment decreased the root-mean-square deviation (RMSD) value and increased the radius of gyration (Rg) value. (**g**) Microscopy image showing the aggregation of Rab11b^-/-^ Neu cells treated with MSC supernatant *in vitro*; n = 5. Scale bar = 20 μm. NS, not significant.

Fig. S6


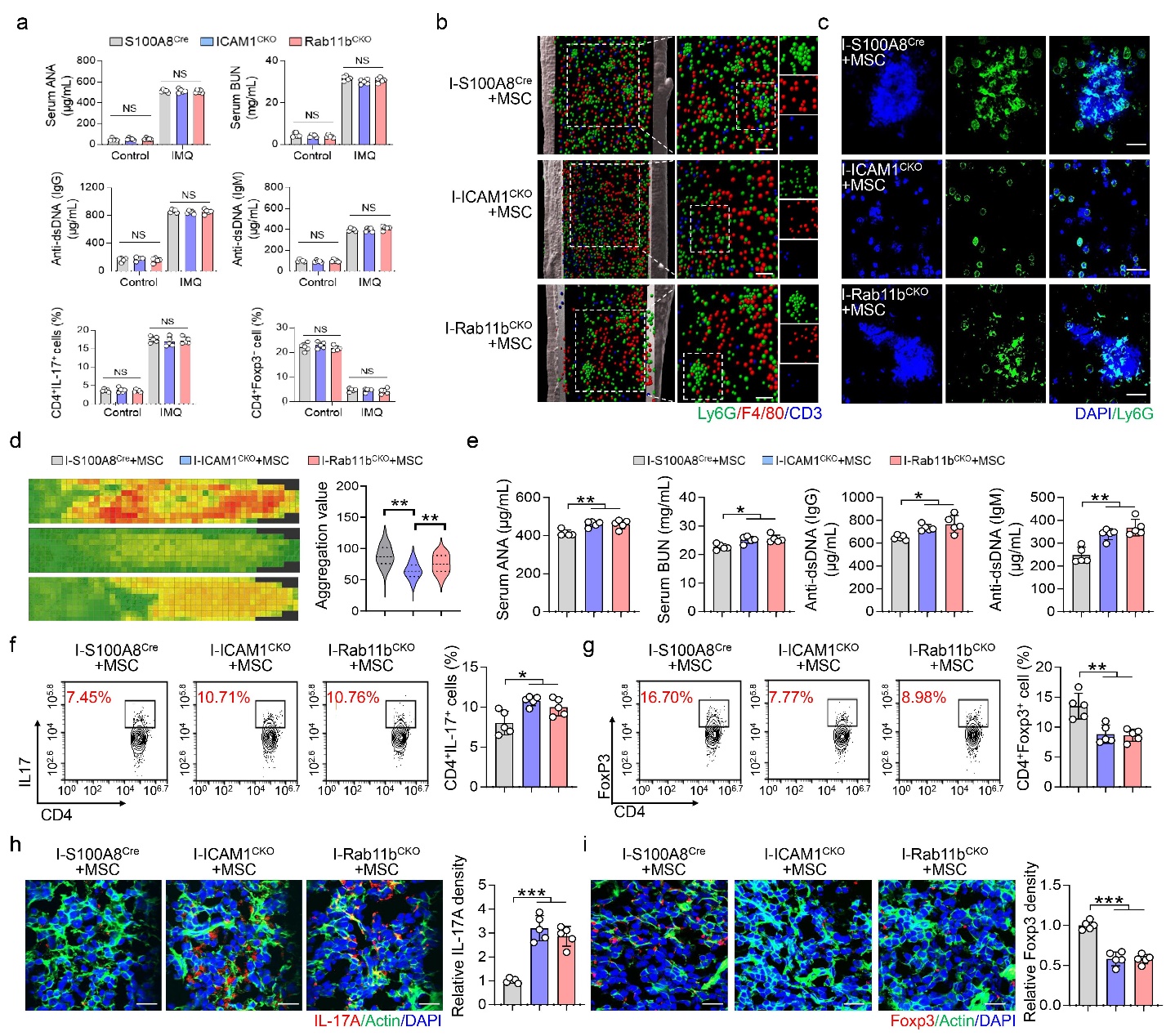


Fig. S6 The efficacy of MSC therapy is impaired in IMQ-induced ICAM1^CKO^ and Rab11b^CKO^ mice.

(**a**) SLE-related indices (ANA, BUN and anti-dsDNA) and the Th17/Treg cell ratio in the spleen were assessed, n = 5 per group. (**b, c**) Tissue transparency experiments (**b**) and bone marrow smears (**c**) revealed that MSC transplantation failed to promote neutrophil aggregation in the bone marrow of IMQ-induced ICAM1^CKO^ (I-ICAM1^CKO^) mice and I-Rab11b^CKO^ mice (n = 3). Scale bar = 200 μm (**b**); Scale bar = 20 μm (**c**). (**d**) HALO proximity analysis revealed that neutrophil aggregation was decreased when MSCs were transplanted into I-ICAM1^CKO^ and I-Rab11b^CKO^ mice (n = 3). (**e**) ELISA revealed that MSC transplantation failed to decrease the concentrations of ANA, BUN and anti-dsDNA in I-ICAM1^CKO^ and I-Rab11b^CKO^ mice (n = 5). (**f, g**) Flow cytometry analysis revealed that MSC treatment failed to decrease the number of Th17 cells and increase the number of Treg cells in I-ICAM1^CKO^ and I-Rab11b^CKO^ mice (n = 5). (**h, i**) Immunofluorescence assays indicated that MSC transplantation failed to inhibit IL-17 expression and promote Foxp3 expression in the spleens of I-ICAM1^CKO^ and I-Rab11b^CKO^ mice (n = 5). Scale bar = 20 μm. **p* < 0.05; ***p* < 0.01; ****p* < 0.001.

Fig. S7


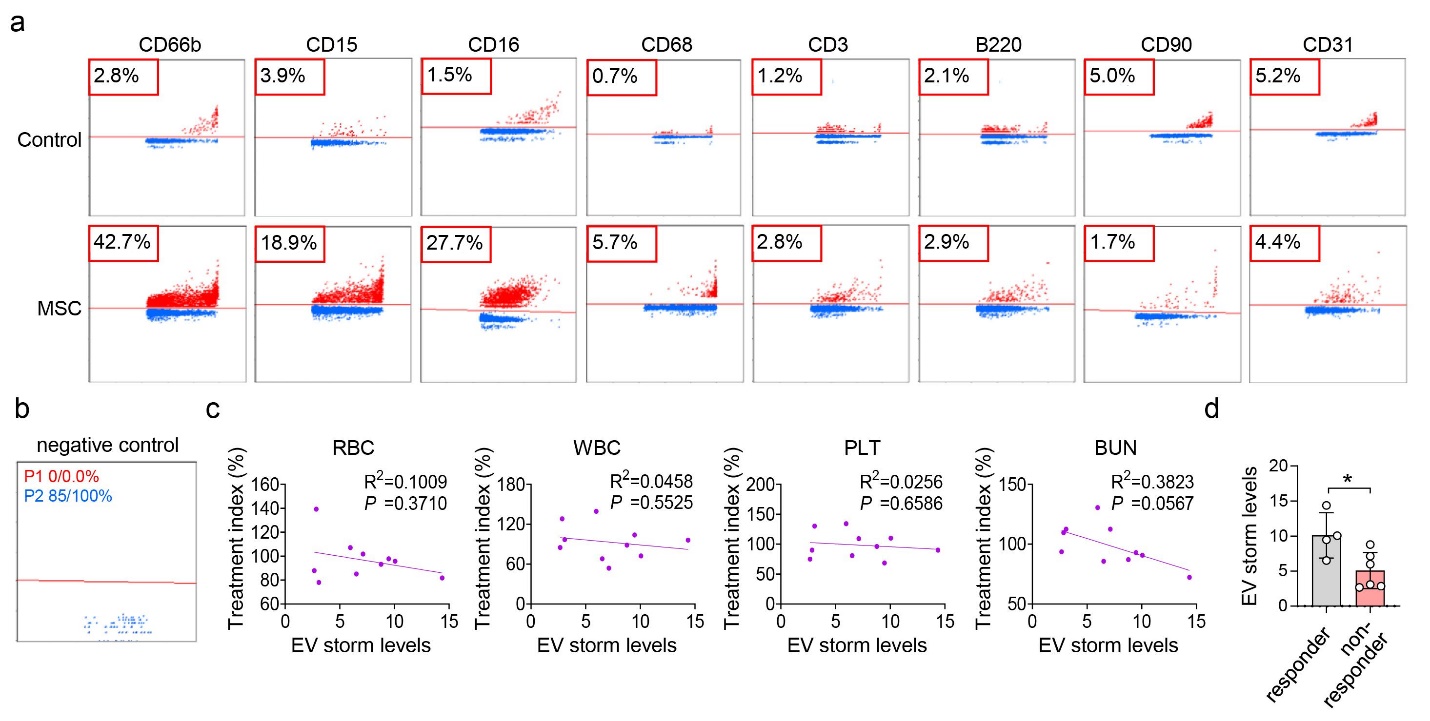


Fig. S7 EV storm analysis from MSC-treated SLE patients.

(**a**) nFCM demonstrated that the number of EVs in the blood of SLE patients highly expressed CD66b, CD15 and CD16 at 4 hours after MSC transplantation but not CD68, CD3, B220, CD90 or CD31, n = 3 per group. (**b**) Negative control for nFCM analysis. (**c**) MSC-induced EV storm rates were not significantly correlated with red blood cell (RBC), white blood cell (WBC), platelet (PLT), or BUN levels (n = 10). The EV storm level is the ratio of (the highest number of EVs among 2, 4, 6, 8 and 12 hours post-MSC transplantation)/(the number of EVs at 0 hours post-MSC transplantation). The treatment index was calculated as (the value of the clinical index after 1 month of MSC transplantation)/(the value before MSC transplantation). (**d**) EV storm rates in responder and nonresponder patients after MSC treatment. NS, not significant; **p* < 0.05.

Fig. S8


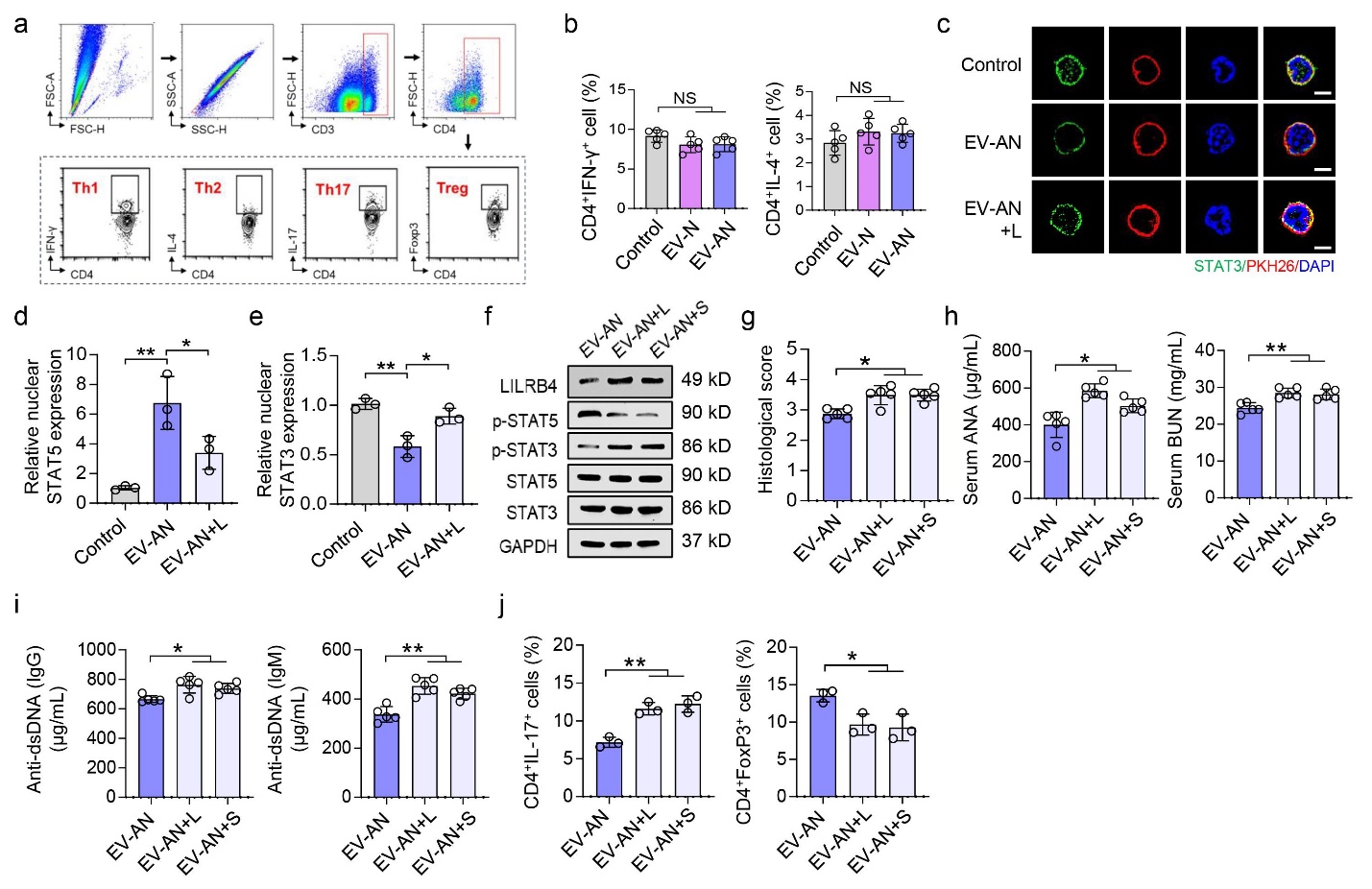


Fig. S8 Aggregated neutrophil-derived EVs induce immune tolerance.

(**a**) Flow cytometry gating strategy for T-cell subpopulations. (**b**) Flow cytometry was used to determine the percentages of Th1 (CD4^+^IFN-γ^+^) and Th2 (CD4^+^IL-4^+^) cells; n = 5 per group. (**c**) Confocal microscopy showing the nuclear translocation of STAT3 in the EV-AN and EV-AN+LILRB4 (EV-AN+L) groups. Scale bar = 5 μm, n = 3. (**d, e**) The relative nuclear STAT5 (**d**) and STAT3 (**e**) expression was analyzed *via* confocal microscopy, n = 3. (**f**) Western blotting was used to detect the expression of LILRB4, p-STAT5 and p-STAT3 in the EV-AN+L and EV-AN+STAT5 phospho-inhibitor (EV-AN+S) groups, n = 3. (**g**) Histological score indices of the kidneys of the EV-AN+L and EV-AN+S groups, n = 5. (**h,** **i**) ELISA revealed that both EV-AN+L and EV-AN+STAT5 increased the levels of serum ANA, BUN and anti-dsDNA *in vivo* (n = 5). (**j**) Flow cytometry was used to detect the percentages of Th17 (CD4^+^IL-17^+^) and Treg (CD4^+^Foxp3^+^) cells in the spleen, n = 3. NS, not significant; **p* < 0.05; ***p* < 0.01.

Table S2. The clinical features of 15 SLE patients

| Features | Results (mean ± SD) |
| --- | --- |
| Age (Years) | 32.7 ± 7.73 |
| SLEDAI | 13.6 ± 5.59 |
| RBC (10^12^/L) | 3.7 ± 0.67 |
| WBC (10^9^/L) | 6.5 ± 2.59 |
| PLT (10^9^/L) | 230.6 ± 94.05 |
| Creatinine (μmol/L) | 102.9 ± 59.34 |
| Albumin (g/L) | 35.5 ± 6.60 |
| Complement C3 (g/L) | 0.8 ± 0.28 |
| Complement C4 (g/L) | 0.2 ± 0.09 |
| Proteinuria (g/24 h) | 6.6 ± 5.29 |
| BUN (mmol/L) | 10.1 ± 5.15 |

SLEDAI, SLE disease activity index; RBC, red blood cell; WBC, white blood cell; PLT, platelet; BUN, blood urea nitrogen.

Table S3. EV numbers in SLE patients after MSC transplantation

| SLE patients | EV number (10^11^/mL) | | | | | | EV storm levels | Follow-up |
| --- | --- | --- | --- | --- | --- | --- | --- | --- |
|  | 0 h | 2 h | 4 h | 6 h | 8 h | 12 h |  |  |
| #1 | 2.12 | 16.48 | 30.46 | 20.35 | 18.45 | 17.64 | 14.37 | Y |
| #2 | 3.46 | 5.47 | 9.86 | 7.65 | 6.58 | 6.02 | 2.85 | Y |
| #3 | 1.33 | 7.69 | 11.68 | 9.58 | 8.63 | 6.45 | 8.78 | N |
| #4 | 3.34 | 4.26 | 8.92 | 8.05 | 7.47 | 4.29 | 2.67 | Y |
| #5 | 3.42 | 16.57 | 24.38 | 20.89 | 17.42 | 11.83 | 7.13 | Y |
| #6 | 5.2 | 14.25 | 24.39 | 31.04 | 25.28 | 19.36 | 5.97 | N |
| #7 | 4.29 | 10.85 | 13.26 | 11.04 | 8.26 | 6.41 | 3.09 | Y |
| #8 | 4.57 | 15.48 | 27.97 | 20.35 | 19.42 | 14.21 | 6.12 | N |
| #9 | 1.03 | 3.25 | 6.05 | 5.37 | 5.19 | 3.29 | 5.87 | Y |
| #10 | 4.16 | 10.36 | 22.42 | 20.35 | 17.35 | 13.35 | 5.39 | N |
| #11 | 4.71 | 8.43 | 11.38 | 13.85 | 10.38 | 9.64 | 2.94 | Y |
| #12 | 3.57 | 8.53 | 6.54 | 3.58 | 3.27 | 4.17 | 2.39 | Y |
| #13 | 1.36 | 8.76 | 13.68 | 11.24 | 10.47 | 5.62 | 10.06 | N |
| #14 | 4.26 | 27.47 | 40.38 | 37.25 | 30.26 | 26.41 | 9.48 | Y |
| #15 | 3.44 | 16.54 | 22.43 | 19.75 | 16.53 | 10.12 | 6.52 | Y |

EV storm levels are calculated as (the highest number of EVs among 2, 4, 6, 8 and 12 hours post-MSC transplantation)/(the number of EVs at 0 hours post-MSC transplantation); Y, yes; N, no.

Table S4. Sample sources and corresponding experimental doses

| **Sample Type** | **Application** | **Amount/Volume/Dose used** |
| --- | --- | --- |
| MSCs | I.V. injection (mouse) | 1×10^6^ cells per mouse |
| Plasma | EV isolation | 200 μL |
| liver | EV isolation | 50 mg |
| Spleen | EV isolation | 50 mg |
| Lung | EV isolation | 50 mg |
| Heart | EV isolation | 50 mg |
| EVs | I.V. injection (mouse) | 1 × 10¹⁰ particles per mouse |
|  | *In vitro* treatment | 1×10^8^ particles/mL |

Table S5. qRT‒PCR primers

| Gene | Forward primer | Reverse primer |
| --- | --- | --- |
| *ICAM1* | ATGCCCAGACATCTGTGTCC | GGGGTCTCTATGCCCAACAA |
| *Rab11b* | TCACCCGCAACGAGTTCAAC | CTGCACCACGGTAGTACGC |
| *GAPDH* | CTGGGCTACACTGAGCACC | AAGTGGTCGTTGAGGGCAATG |

Key resources table

| **Reagent or resource** | **Source** | **Identifier** |
| --- | --- | --- |
| **Antibodies** |  |  |
| PE Anti-Mouse CD63 | Biolegend | 143904 |
| PE Anti-Human CD63 | Biolegend | 353003 |
| PE Anti-Mouse Ly6G | Biolegend | 127608 |
| PE Anti-Mouse F4/80 | Biolegend | 111604 |
| PE Anti-Mouse CD3 | Biolegend | 100206 |
| PE Anti-Mouse IL4 | Biolegend | 504104 |
| PE Anti-Mouse IL-17A | Biolegend | 506904 |
| PE Anti-Mouse FOXP3 | Biolegend | 320008 |
| FITC Anti-Mouse Ly6G | Biolegend | 127606 |
| FITC Anti-Mouse B220 | Biolegend | 103206 |
| FITC Anti-Mouse CD90 | Biolegend | 109008 |
| FITC Anti-Mouse CD31 | Biolegend | 160212 |
| FITC Anti-Mouse IFN-γ | Biolegend | 505806 |
| FITC Anti-Human CD15 | Biolegend | 980502 |
| FITC Anti-Human CD16 | Biolegend | 980112 |
| FITC Anti-Human CD66b | Biolegend | 984102 |
| FITC Anti-Human CD68 | Biolegend | 333805 |
| FITC Anti-Human CD3 | Biolegend | 981002 |
| FITC Anti-Human B220 | Biolegend | 103205 |
| FITC Anti-Human CD90 | Biolegend | 389803 |
| FITC Anti-Human CD31 | Biolegend | 989002 |
| FITC Anti-Human CD3 | Biolegend | 300406 |
| PE/Cyanine7 Anti-Human CD4 | Biolegend | 300512 |
| APC Anti-Human CD25 | Biolegend | 302610 |
| APC/Cyanine7 Anti-Human CD127 | Biolegend | 351348 |
| Brilliant Violet Anti-Human FOXP3 | Biolegend | 320124 |
| PE Anti-Human IL-17A | Biolegend | 512306 |
| FITC Annexin V | Biolegend | 640906 |
| APC Anti-Mouse CD3 | Biolegend | 100236 |
| APC Annexin V | Biolegend | 640920 |
| Percp-Cy5.5 Anti-Mouse CD4 | Biolegend | 116012 |
| Alexa Fluor™ 568 Goat anti-Rat IgG(H+L) | Invitrogen | A11077 |
| Alexa Fluor™ 488 Goat anti-Rat IgG(H+L) | Invitrogen | A11006 |
| Alexa Fluor™ 568 Goat anti-Rabbit IgG(H+L) | Invitrogen | A11036 |
| Alexa Fluor™ 488 Goat anti-Rabbit IgG(H+L) | Invitrogen | A11008 |
| Alexa Fluor™ 568 Goat anti-Mouse IgG(H+L) | Invitrogen | A11004 |
| Alexa Fluor™ 488 Goat anti-Mouse IgG(H+L) | Invitrogen | A11001 |
| Alexa Fluor™ 568 Rabbit anti-Goat IgG(H+L) | Invitrogen | A11079 |
| Alexa Fluor™ 488 Donkey anti-Goat IgG(H+L) | Invitrogen | A11055 |
| Alexa Fluor™ 488 Goat anti-Chicken IgG(H+L) | Invitrogen | A11039 |
| Mouse-IgGκ BP-HRP | Santa Cruz Biotechnology | sc-516102 |
| Goat anti-Rat IgG (H+L) Secondary Antibody, HRP | Thermo Fisher Scientific | 31470 |
| Mouse-IgGκ BP-HRP | Santa Cruz Biotechnology | sc-516142 |
| Mouse anti-Rabbit IgG-B | Santa Cruz Biotechnology | sc-2491 |
| Mouse anti-Rabbit IgG-HRP | Santa Cruz Biotechnology | sc-2357 |
| Mouse anti-Goat IgG-B | Santa Cruz Biotechnology | sc-2489 |
| Mouse anti-Goat IgG-HRP | Santa Cruz Biotechnology | sc-2354 |
| Anti-GAPDH | CST | 5174 |
| Anti-CD63 | Santa Cruz Biotechnology | sc-5275 |
| Anti-CD9 | Santa Cruz Biotechnology | sc-13118 |
| Anti-Alix | Abcam | ab275377 |
| Anti-TSG101 | Abcam | ab125011 |
| Anti-Calnexin | Abcam | ab133615 |
| Anti-Ly6G | CST | 87048 |
| Anti-Ly6C | CST | 37872 |
| Anti-CD3 | CST | 4443 |
| Anti-F4/80 | Abcam | ab300421 |
| Anti-Rab11b | CST | 2414 |
| Anti-Rab27a | CST | 69295 |
| Anti-Rab35 | CST | 9690 |
| Anti-ICAM1 | CST | 4915 |
| Anti-Krt8 | Abcam | ab53280 |
| Anti-Fscn1 | CST | 9269 |
| Anti-LILRB4 | Abcam | ab231813 |
| Anti-p-STAT5 | CST | 4322 |
| Anti-STAT5 | CST | 94205 |
| Anti-p-STAT3 | CST | 9145 |
| Anti-STAT3 | CST | 12640 |
| Anti-Mouse Ly6G Antibody (1A8) | MCE | HY-P990118 |
| Anti-Mouse CD3 Antibody | Abinvivo | B23146201 |
| LEAF™ Purified anti-mouse CD3ε | Biolegend | 100302 |
| LEAF™ Purified anti-mouse CD28 | Biolegend | 102116 |
| **Cytokines and other kits** |  |  |
| Recombinant Murine TNF-α | PeproTech | 315-01a |
| Recombinant Murine IFN-γ | PeproTech | 315-05 |
| Recombinant Murine IL-6 | PeproTech | 216-16-10 |
| Recombinant Human IL-23 | PeproTech | 200-23-10 |
| Recombinant Murine IL-1β | PeproTech | 211-11B-10 |
| Recombinant Mouse TGF-β Protein | R&D | 7666-MB-005 |
| CellMask plasma membrane stain | Invitrogen | C10046 |
| Clodronate Liposomes | Yeasen | 40337ES08 |
| DAPI Medium | Abcam | ab104139 |
| Recombinant TNFα | MCE | HY-P1860 |
| ActinGreen 488 ReadyProbes | Invitrogen | R37110 |
| ActinRed 555 ReadyProbes | Invitrogen | R37112 |
| CellMask DeepRed | Invitrogen | C10046 |
| FoxP3/transcription factor staining kit | Thermo Fisher | 00-5523-00 |
| Pierce MS-Compatible Magnetic IP Kit | Sigma | 90409 |
| Mouse naïve CD4^+^ T-cell isolation kit | Miltenyi Biotec | 130-104-453 |
| Mouse bone marrow neutrophil isolation solution kit | Solarbio | P8550 |
| Tissue clearing kit | NUOHAI | NH-CR-230701 |
| PE Annexin V Apoptosis Detection Kit | Invitrogen | 559763 |
| 7-AAD | Invitrogen | 559925 |
| Annexin V Binding Buffer | Invitrogen | 556454 |
| CFSE Cell Division Tracker Kit | Biolegend | 423801 |
| SC-26196 | MCE | HY-107410 |
| STAT5-IN-1 | MCE | HY-101853 |
