# Supplementary material for: Mesenchymal stromal cells induce neutrophil aggregation and extracellular vesicle storms for systemic lupus erythematosus
Source: Signal Transduct Target Ther. 2025 Oct 13;10:344. doi: 10.1038/s41392-025-02442-1 (PMC12518853; doi:10.1038/s41392-025-02442-1)
Supplement: Supplementary file 3 — Schematic diagram [file 41392_2025_2442_MOESM3_ESM.docx]

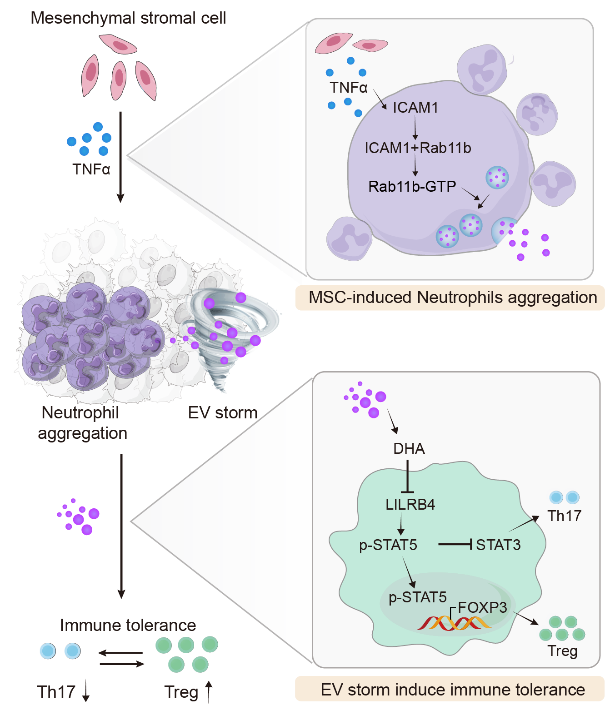


**Schematic diagram:** MSCs induce recipient bone marrow neutrophil aggregation, to generate endogenous EV storms in SLE mice and patients. EV storm is required to induce immune tolerance and rebalance the Th17/Treg cell ratio in MSC-treated SLE mice.
