# Supplementary figures and images for: Mesenchymal stromal cells induce neutrophil aggregation and extracellular vesicle storms for systemic lupus erythematosus
Source: Signal Transduct Target Ther. 2025 Oct 13;10:344. doi: 10.1038/s41392-025-02442-1 (PMC12518853; doi:10.1038/s41392-025-02442-1)

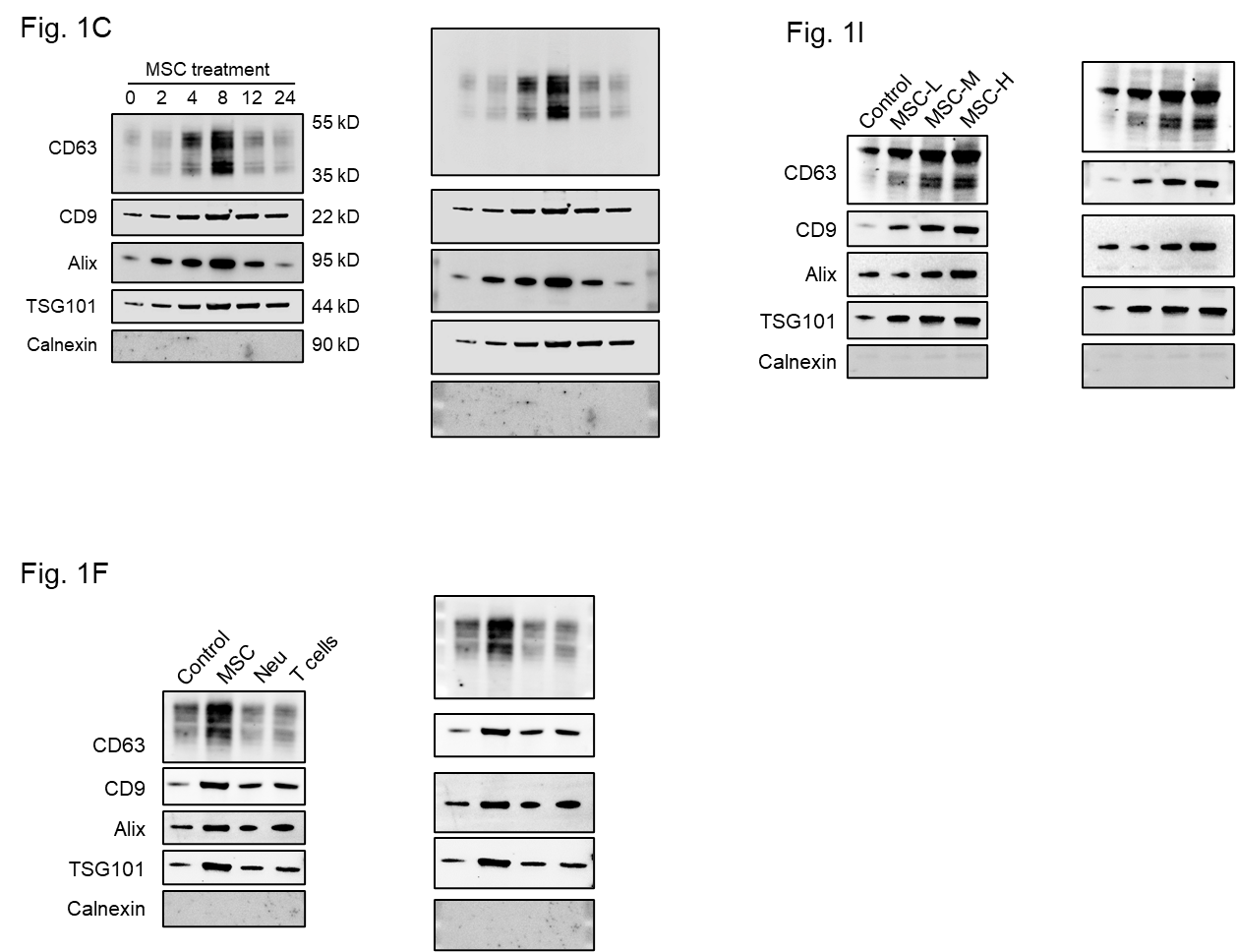

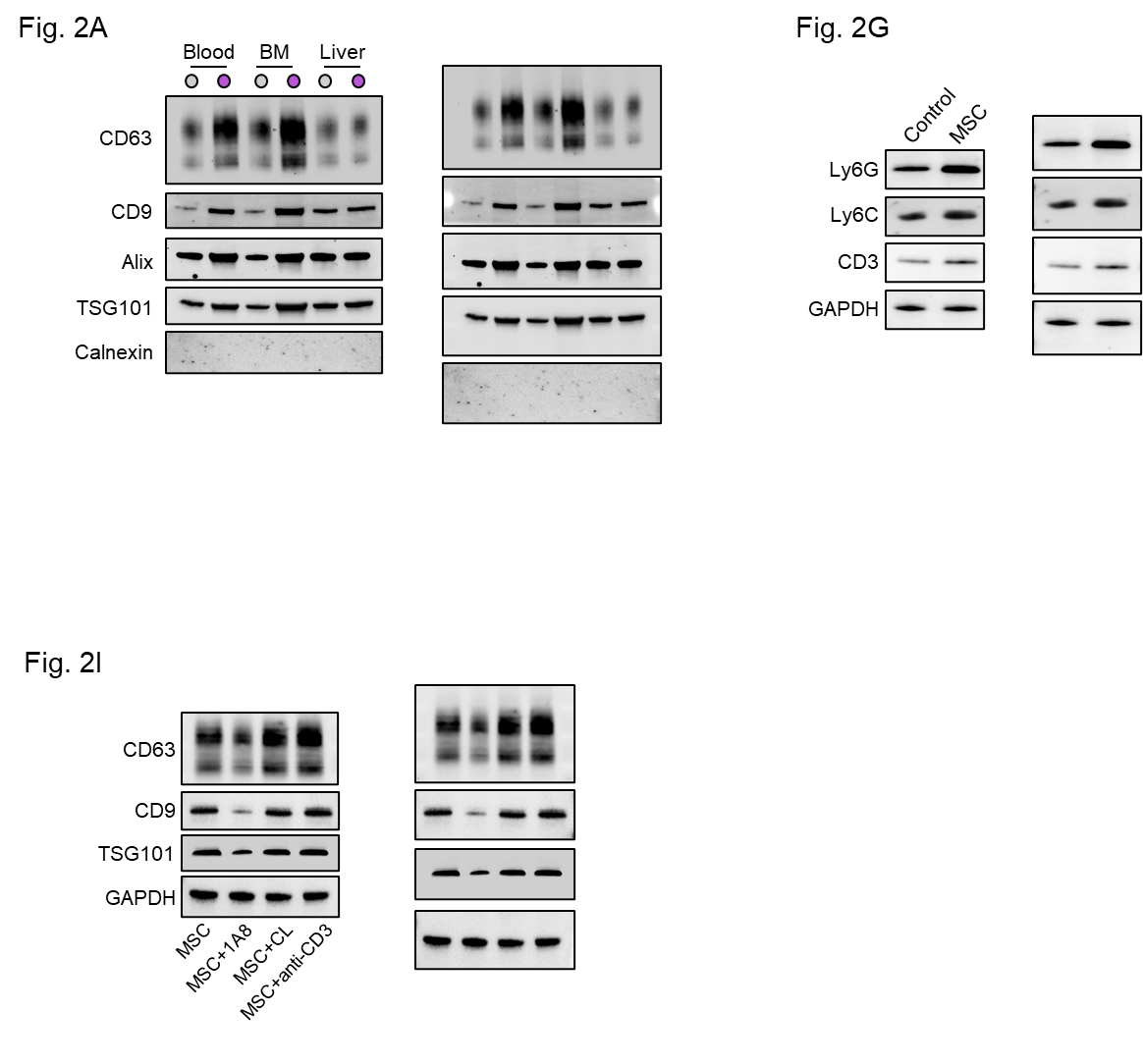

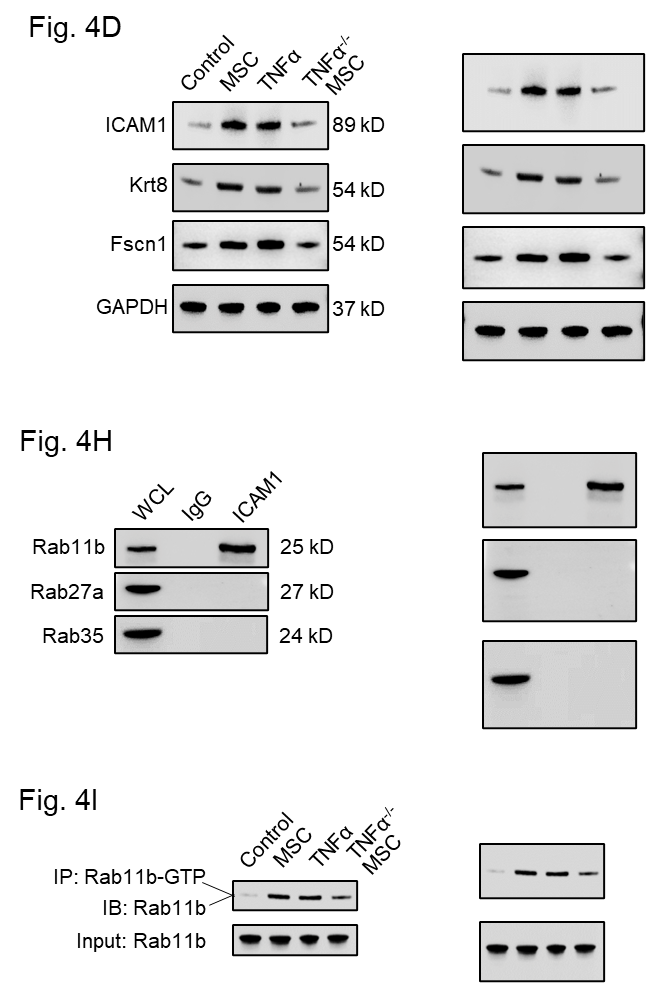

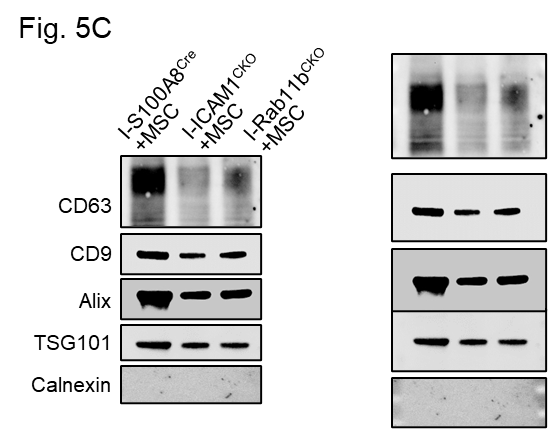

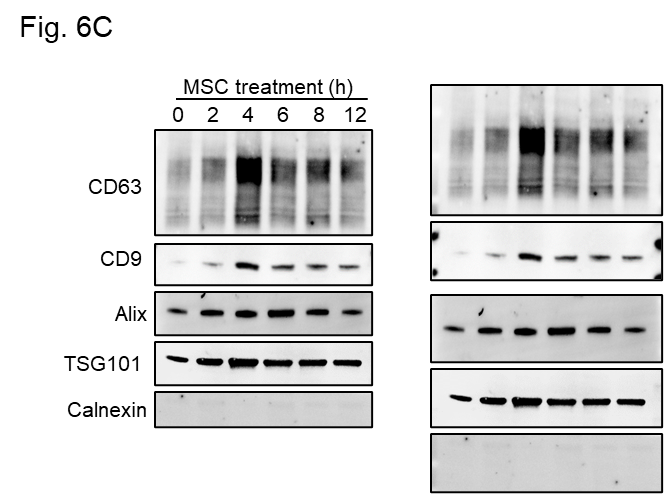

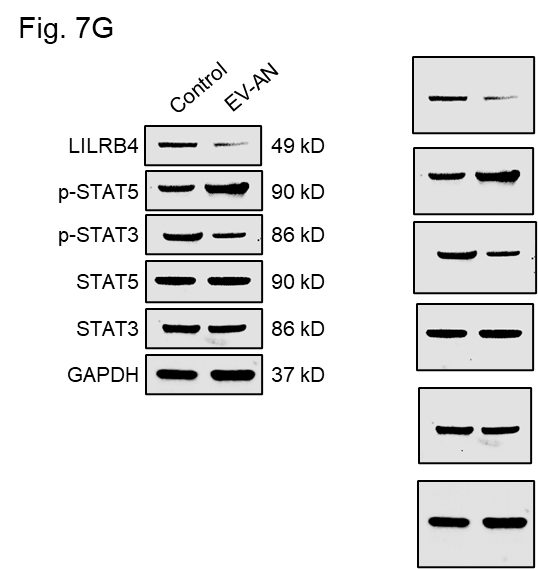

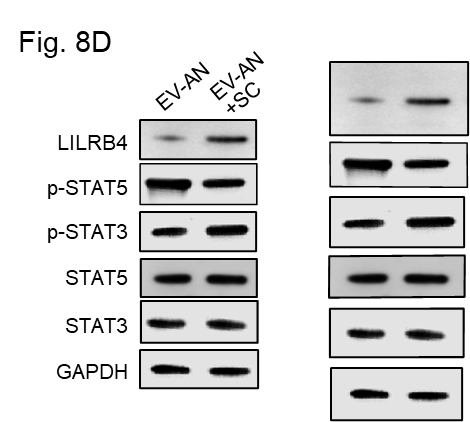

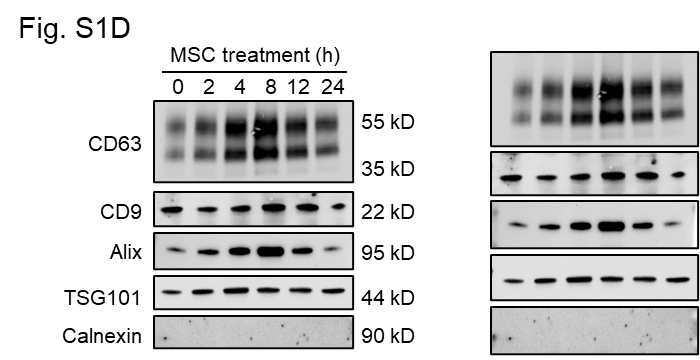

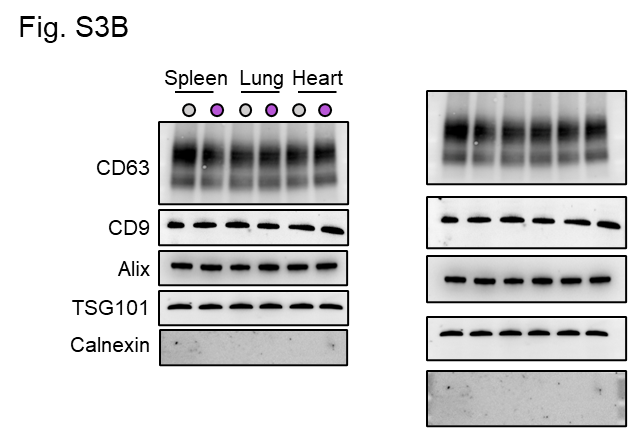

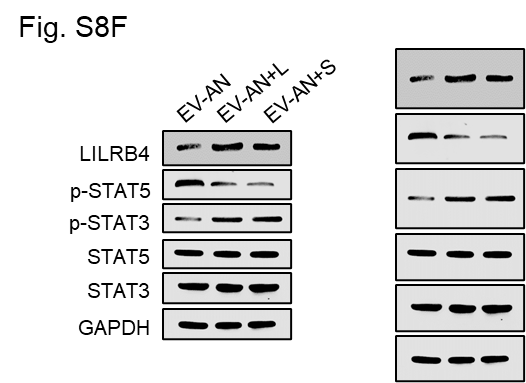

Supplement: Supplementary file 4 — wb raw figures [file 41392_2025_2442_MOESM4_ESM.docx]
